# The extent to which off-patent registered prescription medicines are used for off-label indications in Australia: A scoping review

**DOI:** 10.1371/journal.pone.0261022

**Published:** 2021-12-03

**Authors:** Katrina Howe, Siobhan Bourke, Lloyd Sansom

**Affiliations:** 1 Department of Health Services Research and Policy, Research School of Population Health, College of Health and Medicine, The Australian National University, Canberra, Australia; 2 Clinical and Health Sciences Unit, University of South Australia, Adelaide, Australia; The University of Queensland Medicine Program, AUSTRALIA

## Abstract

**Aim:**

The aim of this scoping review was to determine the extent of off-patent prescription medicine use beyond registered indications in various Australian clinical settings.

**Method:**

The review followed the Joanna Briggs Institute approach and reported using PRISMA Extension for Scoping Reviews. Online databases were used to identify published literature about off-patent registered prescription medicines used for off-label indications in Australian public hospital, community and primary healthcare settings. In addition, empirical data from the Queensland and the South Australian state-wide medicine formularies were screened for the same medication/off-label indication dyads identified in the literature, and other locally approved uses.

**Results:**

Overall, fourteen studies were included, conducted in public hospitals (n = 11), palliative care units (n = 2) and the community setting (n = 1). There were 213 reports extracted from the literature describing off-patent registered prescription medicines used for off-label indications, representing 128 unique medication/off-label indication dyads and 32 different medicines. Of these, just five medication/off-label indication dyads were approved for use on both the Queensland and South Australian state-wide medicine formularies, with 12 others only approved for use in Queensland and 16 others only approved for use in South Australia. Further examination of these state-wide formularies demonstrated that the use of off-patent registered prescription medicines beyond registered indications is more extensive than has been reported to date in the literature. There were 28 additional medication/off-label indication dyads approved on the Queensland state-wide medicine formulary and 14 such examples approved for use in South Australia. Of these, just two medication/off-label indication dyads were approved for use on both formularies.

**Conclusion:**

The extent to which off-patent registered prescription medicines have been repurposed in clinical settings for off-label indications in Australia is greater than previously reported in the literature. Usage and funded availability of certain medication/off-label indication dyads, varies across Australia. These results further expose the two tiered system of medicines regulation in Australia, and its impact on equity of access to medicines. Further research is required to support policy change to encourage submission of registration updates for off-patent prescription medicines.

## Introduction

Medicine repurposing includes a wide range of areas where new therapeutic uses are found for existing medicines. These new therapeutic uses can be: for an additional condition/disease (repositioning), patient population (extension of indication), a different dosage form or route of administration (reformulation) or a combination of these [1, 2].

In Australia, medicines must be assessed for safety, quality and efficacy by the Therapeutic Goods Administration (TGA) and included in the Australian Register of Therapeutic Goods (ARTG) before they can be lawfully supplied [3]. Registration by a sponsor (usually a pharmaceutical company) is necessary for a medicine to be eligible for Australian Government subsidisation via the Pharmaceutical Benefits Scheme (PBS) [3]. In the case of registered prescription medicines that are no longer patent protected (and have lost market exclusivity due to the availability of generic versions), sponsors are unlikely to request registration updates [4]. The use of prescription medicines beyond the limits of their registration or “off-label” is legal [3] and is a form of medicine repurposing where formal regulatory authority approval has not been sought.

Repurposing of existing prescription medicines through off-label use is considered clinically appropriate when no registered alternative treatment is available to a patient, and some scientific and medical evidence exists to support that particular off-label use [5]. In the absence of registration updates for off-patent prescription medicines, individual states and territories conduct their own reviews of safety, efficacy and cost prior to adding a medicine for off-label use to their state-wide medicine formulary or medicine list as an approved treatment [6–8].

However, the use of registered prescription medicines off-label can be problematic for patients. If the cost of the prescription is not PBS subsidised, and the medicine is not available for that off-label use on a state-wide formulary, patients must pay the full price for the medicine [9]. Cost can present a significant financial burden for the patient and their family and may present a significant barrier to accessing clinically appropriate medicine for the patient.

This study aims to determine the extent of off-patent prescription medicine use beyond registered indications in various Australian clinical settings (defined as use for off-label indications).

Several Australian studies have examined off-label prescribing rates. The focus of these studies was the hospital setting, patient admission status or patient population (neonate, paediatric, oncology) or a combination of these, thus limiting their scope. Off-label prescribing rates were found to be 47% within a neonatal intensive care unit [10], 32% for paediatric inpatients at a general hospital [11], 26% for general outpatients [12], 18% for hospitalised oncology patients [13] and 16% (including unregistered medication use) for inpatients at a paediatric teaching hospital [14]. There is evidence from a specialist oncology centre in Australia that rates of off-label prescribing increased by 17% between 2001 and 2008. The most common reason for off-label prescribing was an unregistered indication [15].

To date there have been no other systematic reviews published examining the extent to which off-patent registered prescription medicines are used beyond registered indications in various clinical settings in Australia. Previous systematic reviews have been limited to specific settings such as off-label drug use in oncology [16], in hospitalised paediatric patients [17], in palliative care [18], or using a particular class of medication off-label [19, 20].

To fill this gap, a scoping review was conducted following the approach recommended by the Joanna Briggs Institute (JBI) [21] and reported using PRISMA Extension for Scoping Reviews (PRISMA-ScR) [22]. This methodology enabled a systematic summarisation of the evidence enabling reproducibility whilst providing the flexibility to broadly explore publicly available sources of evidence on this topic. This review utilised a two-step approach to answer: to what extent are off-patent registered prescription medicines used for off-label indications in Australia?

Firstly, an examination of the published academic and grey literature was conducted. Secondly, publicly available empirical data from Australian state-wide medicine formularies were analysed to identify off-patent prescription medicines used beyond registered indications. Lastly, to identify similarities and differences, the findings from the literature were compared with the findings from each state-wide medicine formulary.

This scoping review successfully demonstrates the extent to which off-patent registered prescription medicines have been repurposed in clinical settings and are currently used beyond registered indications in Australia.

## Materials and methods

### Eligibility criteria

A search strategy was developed in order to identify relevant literature, underpinned by key inclusion criteria based on the JBI Population–Concept–Context (PCC) framework (Table 1). Papers were included if they examined prescribing for an off-label indication in an Australian public hospital, outpatient or primary healthcare setting, and where the name of the medicine and the condition/disease being treated were stated. The timeframe included was published academic literature to 6 August 2020 and published grey literature to 14 August 2020. Only studies published in English were included. Studies that aimed to estimate the extended use solely in a broader population (i.e. in children, pregnant women or the elderly), via a new route of administration, or through a new dosage form were excluded to enable the focus to be use for off-label indications.

**Table 1:** Key inclusion criteria.

**P—Population**	Patients treated in Australian public hospital, community and primary healthcare settings with off-patent registered prescription medicines for off-label indications.
**C—Concept**	Evidence of off-patent registered prescription medicines being repurposed in clinical settings for off-label indications in Australia.
**C—Context**	Use of off-patent registered prescription medicines for off-label indications in Australia, where the name of the medicine and the condition/disease being treated are stated.

The review protocol was developed by the authors prior to commencement and is available in S1 Table.

### Search strategy and sources

To identify potentially relevant material for inclusion in this review, MEDLINE (Ovid), Scopus and Web of Science were searched from 15 July 2020 to 6 August 2020 based on the following key concepts and search terms (Table 2) that were developed to capture literature that related to off-label prescribing: “off-label”, “medicine”; “prescribing”; “public hospital”; “primary healthcare”; “Australia”. Boolean operators to narrow, widen and combine literature searches were developed and assistance was sought from an academic librarian.

**Table 2:** Database search terms and synonyms.

off-label OR off label; medicine OR drug OR medicine* OR drug*; prescribing OR prescri* OR prescription OR utilisation OR utilization; public hospital* OR hospital* OR hospital setting*; primary healthcare OR primary medical care OR general practice OR GP setting* OR community setting* OR community healthcare; Australia OR Australia*

The final search strategy for Medline (Ovid) can be found in S2 Table.

A subsequent literature search was performed from 7 August 2020 to 14 August 2020 using a less sensitive search strategy with the terms “off-label” OR “off label” and “Australia” searched to identify additional published academic and grey literature. This search strategy was used in Google Scholar, Informit online, Proquest, websites of Australian Government agencies (.gov.au), Australian academic institutions (.edu.au) and organisations such as The Council of Australian Therapeutic Advisory Groups (CATAG), National Prescribing Service (NPS) Medicinewise (.org.au) were also searched using keywords and synonyms. Google Scholar searches were limited to the first 10 pages.

The references in the bibliography of the papers found in the database search were reviewed using forwards and backwards searches. In Google Scholar, ‘cited by’ was utilised to identify similar resources (forwards search). The reference list of all included records was searched for additional studies not captured by electronic searches (backwards search).

In addition to the literature review, a search of all Australian state and territory health department websites was conducted to identify any medicine formulary lists that were publicly available online. This limited sources to the Queensland Health list of approved medicines (QLD LAM) and the South Australian Medicines Formulary (SAMF), described in more detail in Table 3. It is known that three other jurisdictions (Northern Territory, Tasmania and Western Australia) have state-wide medicine formularies, and hospital-based formularies operate in the ACT, NSW and VIC [23] but none of these were publicly available online.

**Table 3:** Publicly available state-wide medicine formularies.

State-wide medicine formulary	Coverage	Online address	Reference
Queensland Health list of approved medicines (QLD LAM)	Queensland public hospitals and institutions	www.health.qld.gov.au/clinical-practice/guidelines-procedures/medicines/approved-list	[24]
South Australian Medicines Formulary (SAMF)	South Australian public hospitals and health services	https://extapps2.sahealth.sa.gov.au/SAH_DrugFormulary/Account/DrugSearch.aspx	[7]

Medicines on the QLD LAM are available for use in all Queensland Health public hospitals and institutions. The QLD LAM is maintained by Queensland Health with advice provided by the Queensland Health Medicines Advisory Committee (QHMAC). The ‘Formulary notes for the List of Approved Medicines’ document on page 12 states that ‘prescribing off-label is unavoidable and very common’ [25]. It also acknowledges that on occasions, QHMAC knowingly recommends restrictions which specify off-label uses supported by evidence of a reasonable risk benefit profile for the specific therapeutic use.

The medicines on the SAMF are approved for prescribing within South Australian Public Hospitals and Health Services. The SAMF is maintained by South Australia Health with advice provided by the South Australian Formulary Committee (SAFC). For high-cost medicines (costing $10,000 or more per patient per treatment course), advice provided by the South Australian Medicines Evaluation panel (SAMEP). The South Australian Medicines Formulary landing page [7] acknowledges in bold text that ‘Sometimes it may be necessary to use a medicine for an unapproved or “off-label” indication. The CATAG "Guiding Principles for the quality use of off-label medicines" should be utilised when considering use of off-label medicines’ [6].

A copy of the QLD LAM was downloaded in PDF form on 2 October 2020. This was used to investigate the extent to which off-patent registered prescription medicines were approved in Queensland for the same off-label indications as were reported in the literature. Medicines locally approved for use beyond the registered indication were easily identified because the restriction contained one or both of the following statements: ‘When medicines are used in ways other than as specified in the TGA approved product information, documentation and evaluation should be undertaken with reference to QHMAC’s advice in the LAM formulary notes; and the CATAG guiding principles for the quality use of off-label medicines (www.catag.org.au),’ and ‘Where a medicine is not TGA approved, patients should be made fully aware of the status of the medicine and appropriate consent obtained.’

The SAMF was accessed electronically on 28 October 2020 and a similar inquiry was conducted. Medicines on the SAMF were identified as either being available for the PBS or TGA approved use or those provided “off label” [7]. Medicines locally approved for use beyond the registered indication were easily identified because they were either accompanied by a State-wide High Cost Medicines Formulary eligibility form or the restriction contained information about non PBS or other use.

### Evidence screening and selection

Title and abstract screening were completed independently by two reviewers (KH and SB) as follows. The text words contained in the title and abstract, and the index terms used to describe the article were screened. Full text of potentially relevant studies was then independently assessed by the two reviewers to determine if they met the inclusion criteria. Differences of opinion were discussed until a consensus was reached. It was not necessary to ask the opinion of a third reviewer. Material that did not satisfy the eligibility criteria was removed. Again, any conflicts were resolved through discussion.

EndNote [26] was used to manage references identified from Medline (via OVID), Scopus and Web of Science searches. Covidence [27] was used to screen those references. Duplicate copies of imported references were removed. A Microsoft Excel workbook was used to manage and screen references identified from Google Scholar, Informit online, ProQuest, relevant organisations (.gov.au), (.edu.au), (.org.au) and as a result of forwards and backwards searches.

For included references, a full text review was completed independently by KH and SB to check the eligibility criteria was satisfied. Again, any conflicts were resolved through discussion to reach a consensus.

The state-wide medicine formularies were reviewed by one reviewer (KH) to identify any off-patent registered prescription medicines used for off-label indications. In each case, the reviewer confirmed that the medicine satisfied the eligibility criteria.

### Data extraction

One person (KH) carried out the data extraction. Data were extracted from eligible full texts. A data extraction form was designed and the following data items were collected: (i) citation, (ii) objective/s, (iii) participants (characteristics and total number), (iv) concept (off-patent generic drug name, off-label indication for treatment, number of patients treated), (vi) context (treatment setting in Australia). Subsequently, SB performed a check of the extracted data.

Empirical data was extracted by KH from each publicly available state-wide medicine formulary about all medicines used for the same off-label indication that was reported in the literature.

Details of any other off-patent registered prescription medicines (not identified during the literature review) available on the formulary for an off-label indication, were recorded in a separate Microsoft Excel workbook.

A third person (LS) provided assistance when requested to confirm that the state and territory approved use (as described in the entry on the medicine formulary) was indeed off-label for an indication that had not been approved by the TGA in Australia.

### Data analysis and synthesis

A narrative synthesis of the findings from the literature and each publicly available state and territory medicine formulary enabled patterns of use across these sources and relationships in the data to be explored. The extent of the evidence available for use of off-patent prescription medicines for off-label indications was assessed through comparisons between what was found in the literature and data extracted from the QLD LAM and SAMF. A comparison was also made between each publicly available state and territory medicine formulary, to identify similarities and differences with respect to registered prescription medicines approved for off-label indications in Australia, and those uses not reported in the published literature. Variations in terminology used to describe each off-label indication for treatment across all sources was managed through categorisation of very similar indications to facilitate analysis.

## Results

**Fig 1:**
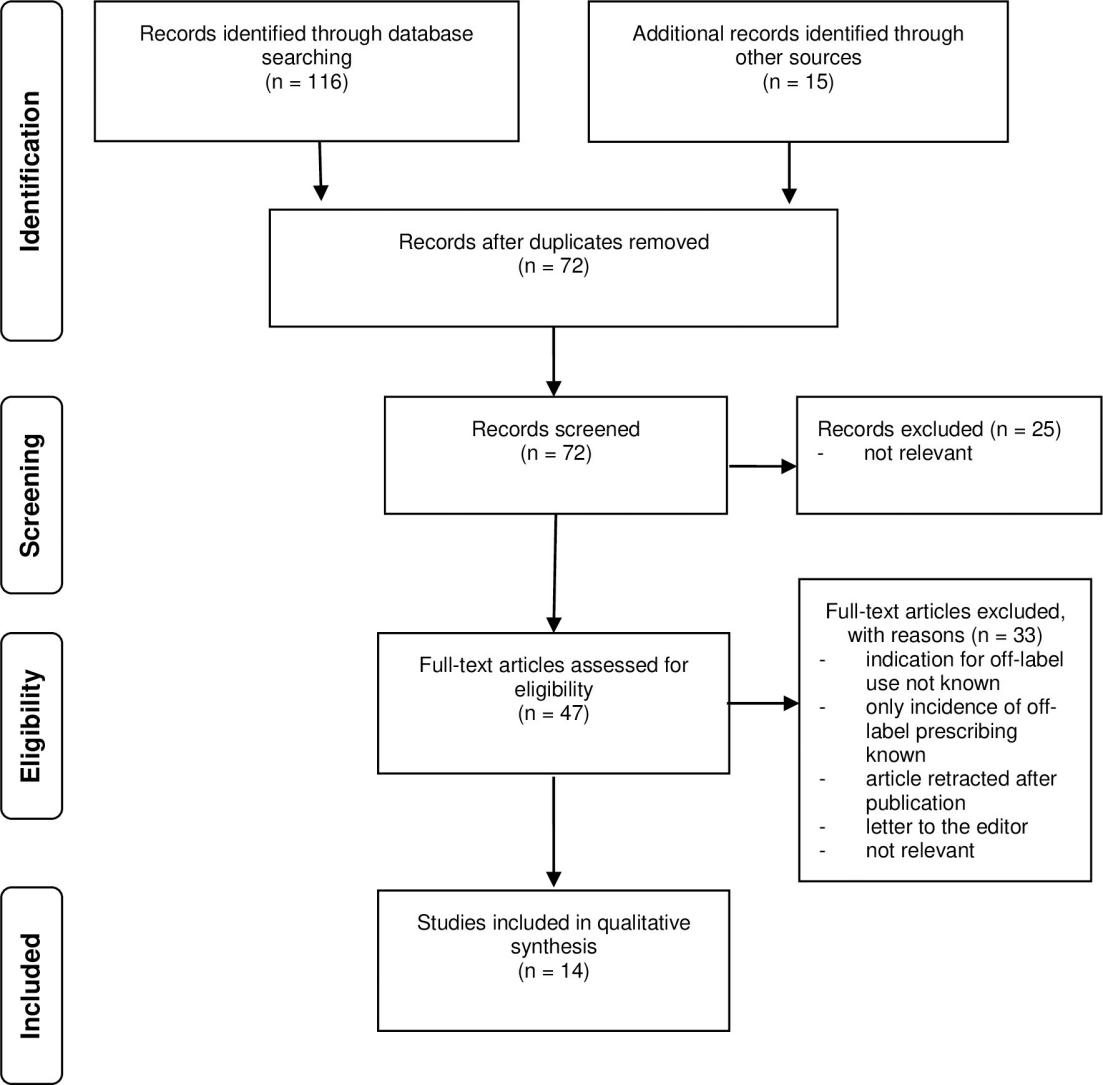
Study flow diagram.

A total of 131 references were returned by the literature review search strategy. The process for identification, screening, assessing eligibility and determining inclusion is described in the study flow diagram (Fig 1).

More information about reporting of results is available at S1 Checklist.

### Review of the literature

Fourteen references [10, 14, 28–39] published between 1999 and 2018, were included.

At least one study was conducted in each Australian state or territory with the exception of the ACT and Tasmania. Three sourced data nationally via online surveys [28–30].

Eleven of these studies reported on off-label use of medicines in one or more Australian public hospitals. Two studies focussed on the off-label use of medicines in palliative care units and one study reported off-label use of ondansetron in pregnancy, presumably, although not stated, in the community setting [31]. None of the literature identified reported on the use of off-patent registered prescription medicines in the primary healthcare or general practice setting.

Fourteen studies focused on certain populations including pregnant women [31], paediatric patients [10, 14, 32]. palliative care patients [29, 30] and patients with dermatological conditions [33], psychiatric disorders [34] and autoimmune or other conditions [30, 35–39].

Overall, this scoping review provides evidence from the literature of the use of 32 off-patent prescription medicines for off-label indications in various clinical settings in Australia. A summary of these results are presented in Table 4.

**Table 4:** Evidence from the literature of off-patent registered prescription medicines used for off-label indications in Australia.

First author and citation	State/ Territory	Setting	Aim	Off-patent registered prescription medicine	Number of off-label indications reported
Newton [28]	Nationally	Palliative care units	To assess the off-label use of nebulised frusemide for breathlessness by Australian physicians working in palliative care using an online survey.	Frusemide	1
O’Connor [30]	Nationally	Public Hospitals	To assess the off-label use of rituximab for off-label indications in Australia, through prospective collection of nationwide data from May 2012 to October 2012	Rituximab	64
To [29]	Nationally	Palliative care units	To document off-label medication/indication dyads and unit policies used by clinicians in Australian palliative care units using a survey (2010).	Clonazepam	5
				Ketamine	1
				Midazolam	3
				Morphine	1
				Octreotide	1
				Olanzapine	2
Brunero [34]	NSW	Public Hospital (Prince of Wales Hospital, Randwick)	To determine the prevalence of psychotropic medication use in general hospitals.	Alprazolam	1
				Amitriptyline	2
				Carbamazepine	1
				Droperidol	1
				Escitalopram	1
				Haloperidol	1
				Lorazepam	1
				Midazolam	2
				Paliperidone	1
				Quetiapine	4
				Risperidone	2
				Sodium Valproate	1
				Temazepam	1
Sharma [39]	NSW	Public Hospital (Westmead)	To characterize the patterns of usage of rituximab, evaluate the appropriateness of prescribing and to establish guidelines for off-label use.	Rituximab	4
Wongseelashote [35]	NT	Public Hospital (Royal Darwin)	To retrospectively evaluate off-label rituximab use in autoimmune diseases in the Top End of the Northern Territory.	Rituximab	23
Butterly [36]	QLD	Public Hospital (Princess Alexandra Hospital, Brisbane)	To review cases of off-label use of rituximab approved by the High Cost Drug Subcommittee at the Princess Alexandra Hospital, Brisbane between 2005 and 2008.	Rituximab	16
Chay [37]	QLD	Public Hospitals (Princess Alexandra and Royal Brisbane and Women’s Hospitals, Brisbane)	To review the off-label use of low-dose rituximab at two university teaching, tertiary referral hospitals, from mid-2008 until the end of 2011.	Rituximab	21
Ong [33]	QLD	Public Hospital (Princess Alexandra Hospital, Brisbane)	To assess the clinical progress of patients receiving high-cost off-label dermatology drugs and the costs of supplying these drugs at a tertiary public hospital in Brisbane, Queensland, between 2002 and 2013.	Cyclosporin	3
				Infliximab	2
				Mycophenolate mofetil	5
				Rituximab	1
				Thalidomide	2
Inglis [38]	SA	Public Hospital (Royal Adelaide Hospital)	To examine the use of non-formulary medicines more than $5000 AUD per year at an Australian public hospital using retrospective audit from January 2015 to December 2015	Infliximab	4
				Intravenous immunoglobulin	2
				Octreotide	1
				Posaconazole	3
				Rituximab	21
O’Donnell [10]	VIC	Public Hospital (Royal Women’s Hospital, Melbourne)	To determine the extent of unlicensed and off-label medication prescribed in a neonatal intensive care unit during a 10-week period.	Theophylline	1
				Aminophylline	1
McD Taylor [32]	VIC	Public Hospitals	To determine the prevalence and nature of off-label and unlicenced medicine use for paediatric patients (aged 0–17 years) in six emergency departments (July 2011 to June 2012, inclusive)	Ondansetron	1
				Cetirizine	1
				Loratadine	1
Colvin [31]	WA	Community setting	To investigate the use of ondansetron in pregnant women giving birth in WA from 2002 to 2005 through a population-based data linkage study.	Ondansetron	1
Turner [14]	WA	Public Hospital (Princess Margaret Hospital for Children, Perth)	To determine the nature and extent of unregistered and off-label drug use in surgical and medical inpatients in a paediatric teaching hospital.	Ondansetron	1
				Clonidine	1

NSW: New South Wales; NT: Northern Territory; QLD: Queensland; SA: South Australia; VIC: Victoria; WA: Western Australia.

Of the 213 reports of off-label prescription medicine use extracted from the literature, 150 reports involved rituximab (70%). Unique medication/off-label indication dyads made up 128 of the 213 reports. Table 5 illustrates the 31 medication/off-label indication dyads reported more than once in the literature. Rituximab was used off-label in 29 of these dyads, and infliximab was used in two. More detail is available in S3 Table.

**Table 5:** Off-patent registered prescription medication/off-label indication dyads reported more than once in the literature.

Number of publications reporting use	Off-patent registered prescription medicine	Off-label indication reported	First author and citation
5	Rituximab	Immune Thrombocytopenia	Butterly [36], Chay [37], Inglis [38], O’Connor [30], Wongseelashote [35]
5	Rituximab	Myositis	Butterly [36], Chay [37], Inglis [38], O’Connor [30], Sharma [39]
5	Rituximab	Pemphigus vulgaris	Chay [37], Inglis [38], Ong [33], Wongseelashote [35], O’Connor [30]
4	Rituximab	Membranous glomerulonephritis	Butterly [36], Inglis [38], O’Connor [30] Wongseelashote [35]
4	Rituximab	Myasthenia gravis	Butterly [36], Inglis [38], O’Connor [30], Wongseelashote [35]
4	Rituximab	Neuromyelitis optica	Chay [37], Inglis [38], O’Connor [30], Wongseelashote [35]
4	Rituximab	Thrombotic thrombocytopenic purpura	Butterly [36], Inglis [38], O’Connor [30], Wongseelashote [35]
3	Rituximab	Autoimmune haemolytic anaemia	Chay [37], O’Connor [30], Wongseelashote [35]
3	Rituximab	Castleman disease	Inglis [38], O’Connor [30], Sharma [39], Wongseelashote [35]
3	Rituximab	Focal segmental glomerulosclerosis	Butterly [36], Inglis [38], Wongseelashote [35]
3	Rituximab	Lupus nephritis	Butterly [36], Chay [37], Wongseelashote [35]
3	Rituximab	Multiple Sclerosis	Chay [37], Inglis [38], O’Connor [30]
3	Rituximab	Systemic Lupus Erythematosus	Inglis [38], O’Connor [30], Wongseelashote [35]
2	Infliximab	Hidradenitis suppurativa	Inglis [38], Ong [33]
2	Infliximab	Pyoderma gangrenosum	Inglis [38], Ong [33]
2	Rituximab	Acquired haemophilia	O’Connor [30], Wongseelashote [35]
2	Rituximab	ANCA vasculitis	O’Connor [30], Wongseelashote [35]
2	Rituximab	Autoimmune encephalitis	Inglis [38], Wongseelashote [35]
2	Rituximab	Catastrophic antiphospholipid syndrome	Chay [37], Wongseelashote [35]
2	Rituximab	Chronic idiopathic urticaria	Chay [37], O’Connor [30]
2	Rituximab	Chronic inflammatory demyelinating polyradiculoneuropathy	O’Connor [30], Wongseelashote [35]
2	Rituximab	Cryoglobulinaemia	Chay [37], Wongseelashote [35]
2	Rituximab	Cryoglobulinaemic vasculitis	Butterly [36], O’Connor [30]
2	Rituximab	Graft-versus-host disease	Inglis [38], O’Connor [30]
2	Rituximab	Grave’s orbitopathy	Inglis [38], O’Connor [30]
2	Rituximab	Haemolytic anaemia	Butterly [36], Inglis [38]
2	Rituximab	Prophylaxis of transplant rejection	Butterly [36], O’Connor [30]
2	Rituximab	Post transplant lymphoproliferative disorder	O’Connor [30], Sharma [39]
2	Rituximab	Sjögren syndrome	Chay [37], O’Connor [30]
2	Rituximab	Stiff person syndrome	Chay [37], O’Connor [30]
2	Rituximab	Systemic sclerosis	Inglis [38], Wongseelashote [35]

### Comparison between the literature and each state-wide medicine formulary

Examination of the QLD LAM, enabled 23 of the 213 reports of off-label registered prescription medicine use extracted from the literature to be matched with a medicine formulary entry. Ten different off-patent registered prescription medicines were approved on the QLD LAM for 17 of the same medication/off-label indication dyads that were reported in the literature. Only two of the medication/off-label indication dyads that were reported in the literature were from studies only conducted in Queensland, with six others reported in studies that collected data nationally and in other states (S4 Table). This suggests that these off-label uses may also be part of clinical practice in the other states that did not have medicine formularies publicly available for examination.

Approximately 30% of the reports (8/23) involved rituximab. Twelve medication/off-label indication dyads reported in the literature were found on the QLD LAM but not the SAMF. One of these involved rituximab. These are shown in Table 6.

**Table 6:** Medication/off-label indication dyads only found on QLD LAM and in the literature.

Off-patent registered prescription medicine	Off-label indication reported	State/territory where study conducted
Clonazepam	Anxiety	Nationally [29]
Intravenous immunoglobulin	Antibody-mediated rejection	SA [38]
Ketamine	Pain	Nationally [29]
Loratadine	Acute allergic reactions and anaphylaxis	VIC [32]
Lorazepam	Agitation	NSW [34]
Ondansetron	Gastroenteritis	VIC [32]
Posaconazole	Acute myeloid leukaemia	SA [38]
Posaconazole	Malignant otitis externa	SA [38]
Posaconazole	Myelodysplastic syndrome	SA [38]
Risperidone	Delirium	NSW [34]
Risperidone	Sundowning/ Delirium	NSW [34]
Rituximab	Thrombotic Thrombocytopenic Purpura	QLD [36], NT [35], Nationally [30], SA [38]

NSW, New South Wales; NT, Northern Territory; QLD, Queensland; SA, South Australia; VIC, Victoria.

Examination of the SAMF enabled 44 of the 213 reports of off-label registered prescription medicine use extracted from the literature to be matched with a medicine formulary entry. Seven different off-patent registered prescription medicines were approved on the SAMF for 21 of the same medication/off-label indication dyads that were reported in the literature. Only five of the medication/off-label indication dyads reported in the literature were from studies conducted in South Australia, with 14 others reported in studies that collected data nationally and in other states (S5 Table), again suggesting widespread off-label use across Australia. Approximately 75% of the reports (32/43) involved rituximab. Sixteen medication/off-label indication dyads reported in the literature were found on the SAMF and not the QLD LAM. Ten of these involved rituximab. These are shown in Table 7.

**Table 7:** Medication/off-label indication dyads only found on SAMF and in the literature.

Off-patent registered prescription medicine	Off-label indication reported	State/territory where study conducted
Droperidol	Nausea/vomiting	NSW [34]
Infliximab	Pyoderma gangrenosum	QLD [33], SA [38]
Morphine	Dyspnoea	Nationally [29]
Quetiapine	Aggression	NSW [34]
Quetiapine	Aggression/ agitation	NSW [34]
Quetiapine	Agitation	NSW [34]
Rituximab	Acquired haemophilia	Nationally [30], NT [35]
Rituximab	ANCA vasculitis	Nationally [30], NT [35]
Rituximab	Antibody-mediated rejection transplant (lung, cardiac, renal)	Nationally [30]
Rituximab	Autoimmune haemolytic anaemia	Nationally [30], NT [35]
Rituximab	Myositis	Nationally [30], NSW [39], QLD [36, 37], SA [38]
Rituximab	Immune Thrombocytopenia	Nationally [30], NT [35], QLD [36, 37], SA [38]
Rituximab	Myasthenia gravis	Nationally [30], NT [35], QLD [36], SA [38]
Rituximab	Pemphigus vulgaris	Nationally [30], NT [35], QLD [33, 37]
Rituximab	Post-bone marrow transplant epstein-barr virus	Nationally [30]
Rituximab	Prophylaxis of transplant rejection	Nationally [30], QLD [36]

NSW, New South Wales; NT, Northern Territory; QLD, Queensland; SA, South Australia.

There were only eight reports in common when the results of examining the QLD LAM and the SAMF against the literature were compared. This represented five medication/off-label indication dyads which were olanzapine for delirium/ agitation (1) and nausea/vomiting (1); ondansetron for nausea/vomiting of pregnancy (1) and post-operative nausea/ vomiting (1) and rituximab for membranous glomerulonephritis (4).

Approvals of rituximab for off-label indications reported in the published literature were more prevalent on the SAMF (75% of all reports) than on the QLD LAM (30% of all reports). Rituximab was only approved for membranous glomerulonephritis and thrombotic thrombocytopenic purpura on the QLD LAM, whereas it was approved for membranous glomerulonephritis as well as acquired haemophilia, anti-neutrophil cytoplasmic antibody vasculitis, antibody-mediated rejection transplant (lung, cardiac, renal), autoimmune haemolytic anaemia, inflammatory myositis, immune thrombocytopenia, myasthenia gravis, pemphigus vulgaris, post-bone marrow transplant epstein-barr virus and prophylaxis of transplant rejection on the SAMF.

One hundred and fifty-four medication/off-label indication dyads reported in the literature were not approved for use on either the QLD LAM or the SAMF. This suggests widespread off-label registered prescription medicine use in clinical settings where the cost of the medicine to the patient is not eligible for subsidisation either locally or nationally.

### Approved off-label indications not reported in the literature

The use of off-patent registered prescription medicines for off-label indications appears to be a much broader than has been reported to date in the literature. Further examination of the QLD LAM and the SAMF evidenced several approved off-label indications for off-patent registered prescription medicines that were not found in the 14 studies included in this review. There were 28 additional off-patent registered prescription medicines approved on the QLD LAM for off-label indications that were not reported in the literature and 14 such examples identified on the SAMF.

Comparison of these additional off-label indications on QLD LAM and SAMF found only two listings in common. These were misoprostol for second line management of primary post-partum haemorrhage/obstetrics and gynaecology indications; and zoledronic acid for paediatric patients with osteogenesis imperfecta/ other primary or secondary osteoporosis, bone lesions.

Excerpts describing the form, strength and restriction that applies to the medication approved for off-label indications are presented in S6 and S7 Tables.

## Discussion

This review provides specific examples of off-patent registered prescription medicines that are used beyond registered indications in various clinical settings in Australia. The extent to which this occurs in the Australian public hospital setting is far greater than has been previously reported in the published literature. Evidence sourced from the published literature and also from publicly available state-wide medicine formularies shows that usage and funded availability of certain medication/off-label indication dyads varies across Australia, raising equity of access issues for patients. These findings therefore bring into question the suitability of the current system of medicines regulation to facilitate Australia-wide access to appropriate medicines [40].

This review provides empirical evidence which highlights that there is broader off-label prescribing occurring in Australian public hospital settings than has been reported in the current literature. Medication/off-label indication dyads that were not reported in the literature are approved on the QLD LAM (28) and the SAMF (14), with only two listings in common. This under reporting of the nature and extent of off-label prescribing may lead to the access and equity problems for patients arising from this practice to persist indefinitely.

Most studies published to date have had a narrower focus than the current review, because they set out to examine off-label prescription medicine use in a particular clinical setting [29] or patient population [32], or involving a particular medicine [36]. The current approach, which utilises both published and empirical evidence to examine the use of off-patent registered prescription medicines for off-label indications across various clinical settings, provides a more comprehensive picture of what is happening in Australia. However, results from general practice and community settings were severely limited by a scarcity of published literature and no publicly available information about reasons for prescribing.

This scoping review has further exposed the extent to which the two tiered system of medicines regulation operating in Australia impacts equity of access to medicines for patients.

The TGA cannot currently insist that registration information, including the Product Information and Consumer Medicine Information, is updated where evidence supports a change in clinical practice. Consequently, guidelines have been produced to assist prescribers to evaluate the appropriateness of the off-label use of prescription medicines. Recommendations include that any off-label use is supported by high-quality medical or clinical evidence, or where research or exceptional use is justified by clinical circumstances [5, 6]. Reliance on guideline recommendations is problematic because individual prescribers need to be prepared to justify and defend their off-label prescribing [41] if the off-label use is not locally approved through a state-wide medicine formulary. In addition, the absence of clinical practice information in Consumer Medicines Information may hinder the ability of a patient to weigh up the risks and benefits of the chosen medicine, and to make informed choices about the treatment being offered.

If high-quality medical or clinical evidence is available for clinical decision making purposes, perhaps these data could also be suitable for registration purposes if submitted. Further research is required in order to examine the current barriers to submission of this evidence in order to improve patient access and support future evidence-based policy change.

The composition of the state-wide medicine formularies used across Australia appear to vary widely. This may be as a result of differing assessment processes, evidentiary requirements for submissions supporting the requested listing, or local economic considerations. There were very few similarities between the medication/off-label indication dyads approved on each state-wide medicine formulary, particularly in the area of rare conditions. Results showed approvals of rituximab for off-label indications reported in the published literature were more prevalent on the SAMF than on the QLD LAM. In addition, a greater total number of medication/off-label indication dyads, were found to have been approved for use on the QLD LAM compared to the SAMF. These findings illustrate the fact that Australian patients with relatively rare auto-immune conditions don’t all have the opportunity to access funded off-label treatment with off-patent registered prescription medicines. It depends on where they live. In addition, in the absence of PBS subsidisation or approved use at the state/ territory level, the full cost of a medicine used for off-label indications must be borne by the patient, presenting a financial burden or a barrier to access for some.

These findings suggest that without addressing the issue of how to consistently evaluate the safety, efficacy and cost impact of off-patent registered prescription medicines that have been repurposed in clinical settings for off-label indications, differing levels of access to medicines is likely to persist across Australia. This issue has been raised in the past [42], and has not been adequately addressed to date.

This scoping review’s contribution to knowledge about drug repurposing in Australia is timely, given the interest shown in this subject through two public consultations during 2020/2021: the parliamentary inquiry into the approval processes for new drugs and novel medical technologies in Australia [43]; and the TGA’s consultation on repurposing of prescription medicines [44].

These outcomes highlight the need for regulatory reform generating more effective policies to facilitate registration updates for off-patent prescription medicines currently used for off-label indications. Registration updates would help to improve the quality use of medicines, and address inequities associated with varying patient access to these medicines across Australia.

This study has several limitations. Some examples of off-label prescribing may have been missed by inclusion of the term ‘off-label’ in the search strategy, in cases where the off-label use of a medicine was reported, but not specified as such. For instance, no clinical or therapeutic guidelines, which would be expected to contain some commonly used examples of off-label prescribing, were identified when searching the published literature. This was also partly due to the fact that some therapeutic guidelines were only accessible via a subscription or membership. Also, when examining the state-wide medicine formularies, not every off-patent registered prescription medicine used for off-label indications could be identified. Only locally approved uses on the SAMF and QLD LAM were captured. Information on prescription medicines used for off-label indications through individual patient use and non-formulary approval medicine requests was not publicly available. Therefore, the off-label use of medicines on state-wide formularies may be under-reported.

Despite these limitations, this review shows that the extent to which off-patent registered prescription medicines are currently used for off-label indications in Australian clinical settings, is far greater than has been previously reported, and may be even larger than these findings demonstrate.

Further research is required to address inconsistencies between locally approved uses for off-patent prescription medicines and the registration label for these medicines to improve the quality use of medicines and equity of access for patients. To assist future policy development and regulatory reform, more research is required to examine barriers affecting the submission of registration updates for off-patent prescription medicines in Australia.

## Conclusion

Off-patent registered prescription medicines have been repurposed in clinical settings and are currently used for off-label indications in Australia. The extent of this clinical practice is greater than has previously been reported in the literature. Some off-label indications are locally approved on state-wide medicine formularies, adversely affecting equitable access to safe and effective off-patent prescription medicines for all Australians. Further research is required in order to support evidence-based policy change to facilitate submission of registration updates for off-patent registered prescription medicines.

## Supporting information

S1 Checklist PRISMA checklist. (DOCX)

S1 Table Scoping review protocol. (DOCX)

S2 Table Full electronic search strategy for Medline (Ovid). (DOCX)

S3 Table Medication/off-label indication dyads extracted from the literature. (DOCX)

S4 Table Medication/off-label indication dyads on QLD LAM and in the literature. (DOCX)

S5 Table Medication/off-label indication dyads on SAMF and in the literature. (DOCX)

S6 Table Medication/off-label indication dyads on QLD LAM not reported in the literature. (DOCX)

S7 Table Medication/off-label indication dyads on SAMF not reported in the literature. (DOCX)
