# Supplementary material for: The extent to which off-patent registered prescription medicines are used for off-label indications in Australia: A scoping review
Source: PLoS One. 2021 Dec 3;16(12):e0261022. doi: 10.1371/journal.pone.0261022 (PMC8641869; doi:10.1371/journal.pone.0261022)
Supplement: S1 Table — (DOCX) [file pone.0261022.s002.docx]

| **Title**  The extent to which off-patent registered prescription medicines are used for off-label indications in Australia: a scoping review protocol | |
| --- | --- |
| **Question**  To what extent are off-patent registered prescription medicines used for off-label indications in Australia? | |
| **Introduction**  Definition of Terms   - Australian Register of Therapeutic Goods (ARTG) –a list of medicines (and other therapeutic goods) approved for supply in Australia and/or approved for export from Australia. - Indication –a specific therapeutic use of the medicine. - Label –the approved registration information on the ARTG - Off-label use –use beyond the limits of registration. - Off-patent medicine –a medicine no longer protected by a legally enforceable intellectual property right. - Patent - a legally enforceable intellectual property right for the medicine. - Pharmaceutical Benefits Scheme (PBS) - an Australian Government program that subsidises the cost of medicines for Australian residents that hold a current Medicare card. - Prescription medicine –a medicine that can only be made available to a patient on the written instruction of an authorised health professional. - Queensland Health list of approved medicines (QLD LAM) - official state-wide formulary for medicines approved for use in all Queensland Health public hospitals and institutions. - Registered –included in the ARTG - South Australian Medicines Formulary (SAMF) - a list of core medicines which are approved for use within South Australian public hospitals and health services. - Therapeutic Goods Administration (TGA) –a group within the Commonwealth Department of Health responsible for the regulation of therapeutic goods. - Therapeutic Goods - products for use in humans in connection with a therapeutic use including: preventing, diagnosing, curing or alleviating a disease, ailment, defect or injury; influencing inhibiting or modifying a physiological process; testing the susceptibility of persons to a disease or ailment; influencing, controlling or preventing conception; testing for pregnancy.   Explanation of need for review  In Australia, medicines must be assessed for safety, quality and efficacy by the Therapeutic Goods Administration (TGA) and included in the Australian Register of Therapeutic Goods (ARTG) before they can be lawfully supplied [1]. The registration label for a medicine describes the indications, dose, route of administration and patient group set out in the TGA-approved Product Information.  Registration by a sponsor (usually a pharmaceutical company) is necessary for a medicine to be eligible for Australian Government subsidisation via the Pharmaceutical Benefits Scheme (PBS) [1]. Prescriptions for public hospital in-patients are not subsidised by the PBS - instead they are funded by state and territory governments [2].  Once a registered medicine has lost patent protection and market exclusivity due to the availability of generic medicines, sponsors tend not invest time or money into submitting registration updates to the TGA [3]. It is not illegal for registered prescription medicines to be used beyond the limits of registration for off-label indications [4].  Off-label prescribing is known to occur when there is no alternative treatment available. Guidelines have been produced to assist prescribers to evaluate the appropriateness of the off-label use of medicines. Recommendations include that any off-label use is supported by high-quality medical or clinical evidence, or where research or exceptional use is justified by clinical circumstances [5], [6].  Off-label prescribing occurs in the primary healthcare, hospital and out-patient settings. Evidence from the international published literature suggests off-label prescribing rates of up to 40% in adults in outpatient settings and up to 90% in hospitalised paediatric patients [5].  In the absence of registration updates for off-patent prescription medicines, individual states and territories conduct their own reviews of safety, efficacy and cost prior to adding a medicine for off-label use to their state-wide medicine formulary or medicine list as an approved treatment [6] [7] [8].  A scoping review will be conducted following the approach recommended by the Joanna Briggs Institute (JBI) [9] and reported using PRISMA Extension for Scoping Reviews (PRISMA-ScR) [10]. This methodology will enable a systematic summarisation of the evidence enabling reproducibility whilst providing the flexibility to broadly explore publicly available sources of evidence on this topic.  This review will examine evidence specifically relating to a sub-set of off-label prescribing in the Australian context. It will focus on identification of registered off-patent prescription medicines used for off-label indications in Australia. To date there have been no other systematic reviews published examining the extent to which off-patent registered prescription medicines are used for off-label indications in various clinical settings in Australia. This review will be the first to present evidence from the published academic and grey literature alongside empirical evidence from publicly available Australian state-wide medicine formularies about registered off-patent prescription medicines used for off-label indications in Australia.  Details of any preliminary searches undertaken  Prior to developing this review protocol, Medline (Ovid) was examined to identify any previously published or currently underway systematic reviews or scoping reviews on a similar or identical topic. No relevant reviews were identified.  Initial attempts were made to access state and territory medicine formulary lists as well as information published on state and territory health department websites about registered off-patent prescription medicines used for off-label indications. Obtaining comprehensive information from all state and territories proved challenging. Only the South Australian Medicines Formulary (SAMF) and the Queensland Health list of approved medicines (QLD LAM) was available publicly online [11], [12]. Western Australia has a state-wide medicines formulary where access is restricted to medicine prescribers working in the Western Australia public health system, along with prescribers working with public patients in private hospitals. Information from the Australian Capital Territory, the Northern Territory, New South Wales, Victoria and Tasmania was not available publicly online.  Overall review objective  The aim of this scoping review is to determine the extent of off-patent prescription medicine use beyond registered indications in various Australian clinical settings. | |
| **Inclusion Criteria** | |
| **i. Population, or participants and conditions of interest** | Patients treated in Australian public hospital, community and primary healthcare settings with off-patent registered prescription medicines for off-label indications. |
| **ii. Concept** | Evidence of off-patent registered prescription medicines being repurposed in clinical settings for off-label indications in Australia. |
| **iii. Context** | Use of off-patent registered prescription medicines for off-label indications in Australia, where the name of the medicine and the condition/disease being treated are stated. |
| **iv. Language** | English (for feasibility) |
| **v. Timeframe** | Published academic literature to 6 August 2020; Published grey literature to 14 August 2020. |
| **Exclusion Criteria**   - off-label use of medicines that are not currently registered in Australia; - off-label use of medicines that are not prescription medicines in Australia; - off-label use of prescription medicines that are not off-patent in Australia; - prescription medicines used off-label for an approved indication in a broader patient population; - languages other than English | |
| **Methods**  The proposed scoping review will be conducted in accordance with the JBI methodology for scoping reviews [9]. This methodology was chosen because it was anticipated that information on this topic in the published academic literature would be limited. It promised to provide the flexibility to explore the breadth of information on this topic from a variety of sources not limited to the published literature, such as grey literature and publicly available Australian state-wide medicine formularies. The methodology would also enable the systematic summarisation of the evidence to confirm the existence of knowledge gaps to be addressed.  The review will be approached from two angles: the first will be to examine the published academic and grey literature; the second will be to analyse empirical data from publicly available Australian state-wide medicine formularies. This dual-angled method will enable a comprehensive review of the literature and empirical evidence available to identify which registered off-patent prescription medicines are used for off-label indications in Australia. | |
| **Search strategy - literature**  The following search strategy will be utilised in this review to locate published academic and grey literature.  Academic literature  A search of Medline (Ovid), Scopus and Web of Science will be undertaken based on keywords and synonyms:  Keywords: off-label, medicine; prescribing; public hospital; primary healthcare; Australia  Synonyms: off-label OR off label; medicine OR drug OR medicine* OR drug*; prescribing OR prescri* OR prescription OR utilisation OR utilization; public hospital* OR hospital* OR hospital setting*; primary healthcare OR primary medical care OR general practice OR GP setting* OR community setting* OR community healthcare; Australia OR Australia*  The text words contained in the title and abstract, and the index terms used to describe the article will be analysed. For included papers, a full text review will be completed.  To check the inclusion and exclusion criteria is satisfied, the reviewers will:   1. search the ARTG here: <https://tga-search.clients.funnelback.com/s/search.html?query=&collection=tga-artg> to confirm that the medicine is approved for supply in Australia. If there are several brands listed then the medicine will be regarded as off-patent; 2. check the latest version of the Poisons Standard here: https://www.tga.gov.au/publication/poisons-standard-susmp to confirm that the medicine as described is classified as a prescription medicine in Australia; 3. for medicines where only one brand is listed on the ARTG    - 1. check to see if the medicine is listed on the PBS and if so, check the F1/F2 formulary allocation here  <https://www.pbs.gov.au/info/industry/pricing/pbs-items/formulary-allocations>. If the drug is in F2 it is likely to be off patent.      2. check the Food and Drug Administration’s (FDA’s) Orange Book of approved drugs here: https://www.accessdata.fda.gov/scripts/cder/daf/. Drugs that are still patent protected should be listed as just one brand with no therapeutic equivalence evaluation.      3. check the FDA’s list of drugs that are off patent, off exclusivity, without a generic here: <https://www.fda.gov/drugs/abbreviated-new-drug-application-anda/list-patent-exclusivity-drugs-without-approved-generic>   Grey literature  The grey literature searches will also be based on key words and synonyms: “off-label” OR “off label” and “Australia”  Websites of Australian Government agencies (.gov.au), Australian academic institutions (.edu.au) and organisations such as Council of Australian Therapeutic Advisory Groups (CATAG), NPS Medicinewise (.org.au) will be searched.  Specific platforms will be included like Informit online, Proquest, and Google Scholar (first 10 pages only) that disseminate information from conferences, reports and working papers. In Google Scholar, ‘cited by’ will be utilised to identify similar resources (forwards search). | |
| **Study/Source of Evidence selection – literature**  One person (KH) will review the published academic and grey literature first in accordance with the search strategy and select material for data extraction. A second person (SB), will perform independent checks to confirm that the search strategy was applied correctly.  The reference list of all included reports and articles will be searched for additional studies not captured by electronic searches (backwards search).  Following the search, all identified citations will be collated and uploaded into Endnote [13] and duplicates removed.  Covidence [14] will be used to screen references identified from MEDLINE (Ovid), Scopus and Web of Science searches.  Title and abstract screening will be completed independently by KH and SB. Conflicts will be resolved and material that does not satisfy requirements of the inclusion and exclusion criteria will be removed.  Full text review will also be completed independently by KH and SB. The full text of selected citations will be assessed in detail against the inclusion criteria. Again, conflicts will be resolved and material that does not satisfy requirements of the inclusion and exclusion criteria will be removed. | |
| **Data Extraction – literature**  Information from published academic and grey literature will be collected in an Excel workbook and will include: date collected, type of information, web-link, citation details, country/origin, background, population, setting, aim, method, results, conclusion, relevance to research question and summary of key findings. | |
| **Search strategy – state-wide medicine formularies**  All publicly available state and territory formularies will be examined in order to identify any registered off-patent prescription medicines used for off-label indications. Restrictions on use in the listings will be checked for statements about medicines locally approved for use beyond the TGA registered indication. | |
| **Study/Source of Evidence selection – state-wide medicine formularies**  The following state-wide medicine formularies will be reviewed by one reviewer (KH) to identify any off-patent registered prescription medicines used for off-label indications and confirm that the medicine satisfies the eligibility criteria:   - Queensland Health list of approved medicines (QLD LAM) [12] - South Australian Medicines Formulary (SAMF) [7] | |
| **Data Extraction – state-wide medicine formularies**  Empirical data will be extracted by KH from each state-wide medicine formulary. Details of off-patent registered prescription medicines used for the same off-label indication that was reported in the literature, will be recorded in a Microsoft Excel workbook.  Details of any other off-patent registered prescription medicines (not identified during the literature review) available on the formulary for an off-label indication, will be recorded in a separate Microsoft Excel workbook.  Data fields will include: prescription drug name, form and strength, and indication(s) for treatment. Checks will confirm the medicine is ARTG listed; a prescription medicine; off-patent and included in the state and territory medicine formulary for an off-label use to treat indications that are not approved by the TGA in Australia.  A third person (LS) will provide assistance when requested to confirm that the state and territory approved use (as described in the entry on the medicine formulary) has not been approved by the TGA in Australia. | |
| **Data Analysis and Presentation – literature and state-wide medicine formularies**  The extracted data will be presented in diagrammatic or tabular form in a manner that aligns to the objectives and questions of this scoping review  The results of the literature search and the study inclusion process will be reported in full in the final scoping review and presented in a PRISMA-ScR flow diagram [15].  Included studies will be presented in a table.  A comparison will be made between each state and territory formulary list, and findings from the search of the literature. This will be presented in a table.  A comparison will also be made to identify any similarities and differences between the publicly available state and territory medicine formularies, with respect to the prescription medicines available for use off-label for indications that are not approved by the TGA in Australia. This will be presented in a table.  Narrative synthesis and descriptive statistics will accompany the results and will describe how the results relate to the review’s objective and questions. | |

**Reference List**

1. Australian Government Department of Health. Australian regulation of prescription medical products 2020 Available from: https://www.tga.gov.au/australian-regulation-prescription-medical-products.

2. Australian Healthcare Associates. Department of Health, PBS Pharmaceuticals in Hospitals Review Report 2017 Available from: https://www.pbs.gov.au/info/reviews/pbs-pharmaceuticals-in-hospitals-review.

3. Seale JP. Off-label prescribing. Medical Journal of Australia. 2014;200(2):65.

4. Day R. Off-label prescribing. Australian prescriber. 2013;36(6):182-3.

5. Gazarian M, Kelly M, McPhee JR, Graudins LV, Ward RL, Campbell TJ. Off‐label use of medicines: consensus recommendations for evaluating appropriateness. Medical journal of Australia. 2006;185(10):544-8.

6. Council of Australian Therapeutic Advisory Groups. Rethinking medicines decision-making in Australian Hospitals. Guiding principles for the quality use of off-label medicines. 2013 [Available from: http://www.catag.org.au/wp-content/uploads/2012/08/OKA9963-CATAG-Rethinking-Medicines-Decision-Making-final1.pdf.

7. South Australian Health. South Australian Medicines Formulary 2021 Available from: https://extapps2.sahealth.sa.gov.au/SAH_DrugFormulary/Account/DrugSearch.aspx.

8. Queensland Health. Guide for requesting changes to the Queensland Health List of Approved Medicines (LAM) 2021 Available from: https://www.health.qld.gov.au/__data/assets/pdf_file/0032/443597/lam-changes-guide.pdf.

9. Peters MDJ, Godfrey C, McInerney P, Munn Z, Tricco A, Khalil H. Chapter 11: Scoping Reviews (2020 version) In: Aromataris E, Munn Z (Editors). JBI Manual for Evidence Synthesis: JBI; 2020[Available from: https://synthesismanual.jbi.global. https://doi.org/10.46658/JBIMES-20-12.

10. Tricco A, Lillie E, Zarin W, O'Brien K, Colquhoun H, Levac D, et al. PRISMA Extension for Scoping Reviews (PRISMA-ScR): Checklist and Explanation. Annals of Internal Medicine. 2018;169(7):467-73.

11. Government of South Australia. SA Medicines Formulary 2020 Available from: https://extapps2.sahealth.sa.gov.au/SAH_DrugFormulary/Account/DrugSearch.aspx.

12. Queensland Government. List of approved medicines (LAM) 2020 Available from: https://www.health.qld.gov.au/clinical-practice/guidelines-procedures/medicines/approved-list.

13. The EndNote Team. EndNote. EndNote X9 ed. Philadelphia, PA: Clarivate; 2013.

14. Covidence systematic review software: Veritas Health Innovation, Melbourne, Australia; [Available from: www.covidence.org.

15. PRISMA Extension for Scoping Reviews (PRISMA-ScR): Checklist and Explanation. Annals of Internal Medicine. 2018;169(7):467-73.
