# Supplementary material for: The extent to which off-patent registered prescription medicines are used for off-label indications in Australia: A scoping review
Source: PLoS One. 2021 Dec 3;16(12):e0261022. doi: 10.1371/journal.pone.0261022 (PMC8641869; doi:10.1371/journal.pone.0261022)
Supplement: S2 Table — (DOCX) [file pone.0261022.s003.docx]

**Q1 Medline**

15 July

| **Set** | **Search Statement** |
| --- | --- |
| 1 | **(off-label or off label).mp. [mp=title, abstract, original title, name of substance word, subject heading word, floating sub-heading word, keyword heading word, organism supplementary concept word, protocol supplementary concept word, rare disease supplementary concept word, unique identifier, synonyms]** |
| 2 | **(medicine or drug or medicine* or drug*).mp. [mp=title, abstract, original title, name of substance word, subject heading word, floating sub-heading word, keyword heading word, organism supplementary concept word, protocol supplementary concept word, rare disease supplementary concept word, unique identifier, synonyms]** |
| 3 | **(prescribing or prescri* or prescription or utilisation or utilization).mp. [mp=title, abstract, original title, name of substance word, subject heading word, floating sub-heading word, keyword heading word, organism supplementary concept word, protocol supplementary concept word, rare disease supplementary concept word, unique identifier, synonyms]** |
| 4 | **(public hospital* or hospital* or hospital setting*).mp. [mp=title, abstract, original title, name of substance word, subject heading word, floating sub-heading word, keyword heading word, organism supplementary concept word, protocol supplementary concept word, rare disease supplementary concept word, unique identifier, synonyms]** |
| 5 | **(primary healthcare or primary medical care or general practice or GP setting* or community setting* or community healthcare).mp. [mp=title, abstract, original title, name of substance word, subject heading word, floating sub-heading word, keyword heading word, organism supplementary concept word, protocol supplementary concept word, rare disease supplementary concept word, unique identifier, synonyms]** |
| 6 | **(Australia or Australia*).mp. [mp=title, abstract, original title, name of substance word, subject heading word, floating sub-heading word, keyword heading word, organism supplementary concept word, protocol supplementary concept word, rare disease supplementary concept word, unique identifier, synonyms]** |
| 7 | **1 and 2 and 3 and 4 and 5 and 6** |
| 8 | **1 and 2 and 3 and 4 and 6** |
| 9 | **1 and 2 and 3 and 5 and 6** |
