# Supplementary material for: The extent to which off-patent registered prescription medicines are used for off-label indications in Australia: A scoping review
Source: PLoS One. 2021 Dec 3;16(12):e0261022. doi: 10.1371/journal.pone.0261022 (PMC8641869; doi:10.1371/journal.pone.0261022)
Supplement: S3 Table — (DOCX) [file pone.0261022.s004.docx]

|  | **Off-patent registered prescription medicine** | **Off-label indication reported** | **Name of first author** | **State/ territory where study conducted** |
| --- | --- | --- | --- | --- |
| 1 | Alprazolam | Dementia | Brunero | NSW |
| 2 | Aminophylline | Prevention or treatment of apnea of prematurity | O'Donnell | VIC |
| 3 | Amitriptyline | Neuropathic pain | Brunero | NSW |
| 4 | Amitriptyline | Pain | Brunero | NSW |
| 5 | Carbamazepine | Severe agitation & psychosis | Brunero | NSW |
| 6 | Cetirizine | Acute allergic reactions and anaphylaxis | Taylor | VIC |
| 7 | Clonazepam | Anxiety | To | National |
| 8 | Clonazepam | Delirium/agitation | To | National |
| 9 | Clonazepam | Distress | To | National |
| 10 | Clonazepam | Pain | To | National |
| 11 | Clonazepam | Sedation | To | National |
| 12 | Clonidine | Analgesia | Turner | WA |
| 13 | Cyclosporin | Chronic idiopathic urticaria | Ong | QLD |
| 14 | Cyclosporin | Lichen planopilaris of scalp and eyebrows | Ong | QLD |
| 15 | Cyclosporin | Vulvar lichen sclerosus | Ong | QLD |
| 16 | Droperidol | Nausea/vomiting | Brunero | NSW |
| 17 | Escitalopram | Bipolar affective disorder | Brunero | NSW |
| 18 | Frusemide | Intractable breathlessness | Newton | National |
| 19 | Haloperidol | Agitation | Brunero | NSW |
| 20 | Infliximab | Behcet’s disease | Inglis | SA |
| 21 | Infliximab | Hidradenitis suppurativa | Inglis | SA |
| 22 | Infliximab | Hidradenitis suppurativa | Ong | QLD |
| 23 | Infliximab | Pyoderma gangrenosum | Ong | QLD |
| 24 | Infliximab | Pyoderma grangrenosum | Inglis | SA |
| 25 | Infliximab | Sarcoidosis | Inglis | SA |
| 26 | Intravenous immunoglobulin | Antibody-mediated rejection | Inglis | SA |
| 27 | Intravenous immunoglobulin | Peripheral neuropathy | Inglis | SA |
| 28 | Ketamine | Pain | To | National |
| 29 | Loratadine | Acute allergic reactions and anaphylaxis | Taylor | VIC |
| 30 | Lorazepam | Agitation | Brunero | NSW |
| 31 | Midazolam | Agitation | Brunero | NSW |
| 32 | Midazolam | Anxiety | To | National |
| 33 | Midazolam | Dyspnoea | To | National |
| 34 | Midazolam | Pain | To | National |
| 35 | Midazolam | Severe agitation | Brunero | NSW |
| 36 | Morphine | Dyspnoea | To | National |
| 37 | Mycophenolate mofetil | Atopic dermatitis | Ong | QLD |
| 38 | Mycophenolate mofetil | Chronic plaque psoriasis | Ong | QLD |
| 39 | Mycophenolate mofetil | Pemphigus foliaceous | Ong | QLD |
| 40 | Mycophenolate mofetil | Pemphigus vulgaris | Ong | QLD |
| 41 | Mycophenolate mofetil | Urticarial dermatitis | Ong | QLD |
| 42 | Octreotide | Nausea/vomiting | To | National |
| 43 | Octreotide | Thyroid cancer | Inglis | SA |
| 44 | Olanzapine | Delirium/agitation | To | National |
| 45 | Olanzapine | Nausea/vomiting | To | National |
| 46 | Ondansetron | Gastroenteritis | Taylor | VIC |
| 47 | Ondansetron | Nausea and Vomiting of pregnancy | Colvin | WA |
| 48 | Ondansetron | Post-operative nausea and vomiting | Turner | WA |
| 49 | Paliperidone | Anxiety | Brunero | NSW |
| 50 | Posaconazole | Acute myeloid leukaemia | Inglis | SA |
| 51 | Posaconazole | Malignant otitis exterma | Inglis | SA |
| 52 | Posaconazole | Myelodysplastic syndrome | Inglis | SA |
| 53 | Quetiapine | Aggression | Brunero | NSW |
| 54 | Quetiapine | Aggression/ agitation | Brunero | NSW |
| 55 | Quetiapine | Agitation | Brunero | NSW |
| 56 | Quetiapine | Dementia | Brunero | NSW |
| 57 | Risperidone | Delirium | Brunero | NSW |
| 58 | Risperidone | Sundowning/ Delirium | Brunero | NSW |
| 59 | Rituximab | Acquired haemophilia | O'Connor | National |
| 60 | Rituximab | Acquired haemophilia A | Wongseelashote | NT |
| 61 | Rituximab | Acquired von Willebrand factor deficiency | Wongseelashote | NT |
| 62 | Rituximab | Acute inflammatory demyelinating polyneuropathy (AIDP) | O'Connor | National |
| 63 | Rituximab | Acute lymphoblastic leukaemia | O'Connor | National |
| 64 | Rituximab | acute organ rejection | Sharma | NSW |
| 65 | Rituximab | ANCA vasculitis | O'Connor | National |
| 66 | Rituximab | ANCA-associated vasculitis | Wongseelashote | NT |
| 67 | Rituximab | Antibody-mediated rejection transplant (lung, cardiac, renal) | O'Connor | National |
| 68 | Rituximab | Anti-glutamic acid decarboxylase cerebellar ataxia | Chay | QLD |
| 69 | Rituximab | Anti-MuSK antibody-positive Myasthenia gravis | Butterly | QLD |
| 70 | Rituximab | Antiphospholipid syndrome | O'Connor | National |
| 71 | Rituximab | Antiphospholipid syndrome and Systemic Lupus Erythematosus | Chay | QLD |
| 72 | Rituximab | Aplastic anaemia | O'Connor | National |
| 73 | Rituximab | Atopic eczema | Chay | QLD |
| 74 | Rituximab | Autoimmune autonomic neuropathy | Inglis | SA |
| 75 | Rituximab | Autoimmune cytopenia | O'Connor | National |
| 76 | Rituximab | Autoimmune encephalitis | Inglis | SA |
| 77 | Rituximab | Autoimmune encephalopathy | Wongseelashote | NT |
| 78 | Rituximab | Autoimmune haemolytic anaemia | O'Connor | National |
| 79 | Rituximab | Autoimmune haemolytic anaemia | Wongseelashote | NT |
| 80 | Rituximab | Autoimmune haemolytic anaemia | Chay | QLD |
| 81 | Rituximab | Autoimmune hepatitis | Chay | QLD |
| 82 | Rituximab | Autoimmune neuroretinitis | O'Connor | National |
| 83 | Rituximab | Autoimmune-mediated necrotising myopathy | Inglis | SA |
| 84 | Rituximab | Autonomic neuropathy secondary to systemic lupus erythematosus | Butterly | QLD |
| 85 | Rituximab | Behcet scleritis | O'Connor | National |
| 86 | Rituximab | Buerger-like vasculitis | O'Connor | National |
| 87 | Rituximab | Castleman disease | Inglis | SA |
| 88 | Rituximab | Castleman disease | O'Connor | National |
| 89 | Rituximab | Castleman’s disease | Sharma | NSW |
| 90 | Rituximab | Catastrophic antiphospholipid syndrome | Wongseelashote | NT |
| 91 | Rituximab | Catastrophic antiphospholipid syndrome | Chay | QLD |
| 92 | Rituximab | Chronic fatigue syndrome | O'Connor | National |
| 93 | Rituximab | Chronic idiopathic urticaria | Chay | QLD |
| 94 | Rituximab | Chronic idiopathic urticaria | O'Connor | National |
| 95 | Rituximab | Chronic infammatory demyelinating polyneuropathy | Inglis | SA |
| 96 | Rituximab | Chronic inflammatory demyelinating polyradiculoneuropathy | O'Connor | National |
| 97 | Rituximab | Chronic inflammatory demyelinating polyradiculoneuropathy | Wongseelashote | NT |
| 98 | Rituximab | Chronic seronegative polyarthritis | Wongseelashote | NT |
| 99 | Rituximab | Cicatricial pemphigoid | O'Connor | National |
| 100 | Rituximab | Cold agglutinin disease | O'Connor | National |
| 101 | Rituximab | Cryoglobulinaemic vasculitis | O'Connor | National |
| 102 | Rituximab | Cryoglobulinaemic vasculitis | Butterly | QLD |
| 103 | Rituximab | cryoglobulinaemic vasculitis | Chay | QLD |
| 104 | Rituximab | Diabetes (anti-insulin antibodies) | O'Connor | National |
| 105 | Rituximab | Eczema | O'Connor | National |
| 106 | Rituximab | Eosinophilic polyangiitis | O'Connor | National |
| 107 | Rituximab | Focal segmental glomerulosclerosis | Inglis | SA |
| 108 | Rituximab | Focal segmental glomerulosclerosis | Wongseelashote | NT |
| 109 | Rituximab | Focal segmental sclerosing glomerulosclerosis | Butterly | QLD |
| 110 | Rituximab | Glomerulonephritis/Nephrotic syndrome | O'Connor | National |
| 111 | Rituximab | Goodpasture disease | O'Connor | National |
| 112 | Rituximab | Graft-versus-host disease | Inglis | SA |
| 113 | Rituximab | Graft-versus-host-disease post-Bone Marrow Transplant | O'Connor | National |
| 114 | Rituximab | Grave’s orbitopathy | Inglis | SA |
| 115 | Rituximab | Graves’ ophthalmopathy | O'Connor | National |
| 116 | Rituximab | Haemolytic anaemia | Butterly | QLD |
| 117 | Rituximab | Haemolytic anaemia | Inglis | SA |
| 118 | Rituximab | Haemophilia | Inglis | SA |
| 119 | Rituximab | HBV liver failure for transplant | O'Connor | National |
| 120 | Rituximab | Hepatitis C associated mixed cryoglobulinaemia | Wongseelashote | NT |
| 121 | Rituximab | Hereditary haemophilia | O'Connor | National |
| 122 | Rituximab | Idiopathic thrombocytopaenia purpure | Inglis | SA |
| 123 | Rituximab | Idiopathic thrombocytopenic purpura | Butterly | QLD |
| 124 | Rituximab | IgG4-related sclerosing disease | O'Connor | National |
| 125 | Rituximab | IgM paraproteinemic polyneuropathy | Inglis | SA |
| 126 | Rituximab | Immune Thrombocytopenia | Chay | QLD |
| 127 | Rituximab | Immune Thrombocytopenia | O'Connor | National |
| 128 | Rituximab | Immune thrombocytopenic purpura | Wongseelashote | NT |
| 129 | Rituximab | Inflammatory myopathy | Wongseelashote | NT |
| 130 | Rituximab | Inflammatory orbitopathy | O'Connor | National |
| 131 | Rituximab | Interstitial lung disease | O'Connor | National |
| 132 | Rituximab | Lambert–Eaton syndrome | O'Connor | National |
| 133 | Rituximab | Limbic encephalitis | Chay | QLD |
| 134 | Rituximab | Lupus nephritis | Chay | QLD |
| 135 | Rituximab | Lupus nephritis | Wongseelashote | NT |
| 136 | Rituximab | Lupus nephritis (class IV) | Butterly | QLD |
| 137 | Rituximab | Lymphoma unspecified | O'Connor | National |
| 138 | Rituximab | MALT lymphoma | O'Connor | National |
| 139 | Rituximab | Mantle cell lymphoma | O'Connor | National |
| 140 | Rituximab | Membranous glomerulonephritis | Inglis | SA |
| 141 | Rituximab | Membranous glomerulonephritis | O'Connor | National |
| 142 | Rituximab | Membranous nephritis | Butterly | QLD |
| 143 | Rituximab | Membranous nephropathy | Wongseelashote | NT |
| 144 | Rituximab | Minimal change disease | Wongseelashote | NT |
| 145 | Rituximab | Minimal change nephrotic syndrome | O'Connor | National |
| 146 | Rituximab | Mixed connective tissue disease | O'Connor | National |
| 147 | Rituximab | Mucocutaneous lichen planus | O'Connor | National |
| 148 | Rituximab | Mucous membrane pemphigoid | Chay | QLD |
| 149 | Rituximab | Multiple Sclerosis | Chay | QLD |
| 150 | Rituximab | Multiple Sclerosis | Inglis | SA |
| 151 | Rituximab | Multiple sclerosis | O'Connor | National |
| 152 | Rituximab | Multiple sclerosis and ankylosing spondylitis | Chay | QLD |
| 153 | Rituximab | Myaesthenia gravis | O'Connor | National |
| 154 | Rituximab | Myasthenia gravis | Inglis | SA |
| 155 | Rituximab | Myasthenia gravis | Butterly | QLD |
| 156 | Rituximab | Myasthenia gravis | Wongseelashote | NT |
| 157 | Rituximab | Myositis | Chay | QLD |
| 158 | Rituximab | Myositis (dermatomyositis) | Butterly | QLD |
| 159 | Rituximab | Myositis (inflammatory) | Inglis | SA |
| 160 | Rituximab | Myositis (polymyositis) | Sharma | NSW |
| 161 | Rituximab | Myositis/polymyositis/dermatomyositis | O'Connor | National |
| 162 | Rituximab | Neuromyelitis optica | O'Connor | National |
| 163 | Rituximab | Neuromyelitis optica | Wongseelashote | NT |
| 164 | Rituximab | Neuromyelitis optica | Chay | QLD |
| 165 | Rituximab | Neuromyelitis optica and Systemic Lupus Erythematosus | Chay | QLD |
| 166 | Rituximab | Neuromyelitis optica spectrum disorder | Inglis | SA |
| 167 | Rituximab | Neuropathy unspecified | O'Connor | National |
| 168 | Rituximab | Opsoclonus myoclonus ataxia | O'Connor | National |
| 169 | Rituximab | Other vasculitis | O'Connor | National |
| 170 | Rituximab | Panarteritis nodos | O'Connor | National |
| 171 | Rituximab | Paraneoplastic and Autoimmune encephalitis | O'Connor | National |
| 172 | Rituximab | Pemphigus | O'Connor | National |
| 173 | Rituximab | Pemphigus vulgaris | Inglis | SA |
| 174 | Rituximab | Pemphigus vulgaris | Chay | QLD |
| 175 | Rituximab | Pemphigus vulgaris | Ong | QLD |
| 176 | Rituximab | Pemphigus vulgaris | Wongseelashote | NT |
| 177 | Rituximab | Post- Epstein-Barr Virus encephalopathy | O'Connor | National |
| 178 | Rituximab | Post transplant hemolytic uremic syndrome recurrence | Butterly | QLD |
| 179 | Rituximab | Post-bone marrow transplant epstein-barr virus | O'Connor | National |
| 180 | Rituximab | Post-transplant lymphoproliferative disease | Sharma | NSW |
| 181 | Rituximab | Presumed Hashimoto’s encephalitis | Chay | QLD |
| 182 | Rituximab | Prophylaxis of transplant rejection | O'Connor | National |
| 183 | Rituximab | Post-transplant lymphoproliferative disease (liver, renal, cardiac) | O'Connor | National |
| 184 | Rituximab | Rasmussen syndrome | O'Connor | National |
| 185 | Rituximab | Recurrence of Focal Glomerular Sclerosis | O'Connor | National |
| 186 | Rituximab | Recurrent parotitis | Wongseelashote | NT |
| 187 | Rituximab | Renal transplant (B cell-positive cross match) | Butterly | QLD |
| 188 | Rituximab | Scleroderma | O'Connor | National |
| 189 | Rituximab | Shrinking lung syndrome secondary to systemic lupus erythematosis lupus nephritis (class III) | Butterly | QLD |
| 190 | Rituximab | Sjögren syndrome | O'Connor | National |
| 191 | Rituximab | Sjögren syndrome | Chay | QLD |
| 192 | Rituximab | Small intestinal vasculitis | O'Connor | National |
| 193 | Rituximab | Stiff person syndrome | Chay | QLD |
| 194 | Rituximab | Stiff person syndrome | O'Connor | National |
| 195 | Rituximab | Subacute cerebellar degeneration (anti-Yo antibody positive) | Butterly | QLD |
| 196 | Rituximab | Systemic lupus erythematosus | Inglis | SA |
| 197 | Rituximab | Systemic Lupus Erythematosus | O'Connor | National |
| 198 | Rituximab | Systemic Lupus Erythematosus | Wongseelashote | NT |
| 199 | Rituximab | Systemic sclerosis | Inglis | SA |
| 200 | Rituximab | Systemic sclerosis | Wongseelashote | NT |
| 201 | Rituximab | Thrombotic thrombocytopenic purpura | Butterly | QLD |
| 202 | Rituximab | Thrombotic thrombocytopenic purpura | Wongseelashote | NT |
| 203 | Rituximab | Thrombotic thrombocytopenic purpura | O'Connor | National |
| 204 | Rituximab | Thrombotic thrombocytopenic purpure | Inglis | SA |
| 205 | Rituximab | Thyroid acropathy | Butterly | QLD |
| 206 | Rituximab | Transverse myelitis | Wongseelashote | NT |
| 207 | Rituximab | Ulcerative colitis | O'Connor | National |
| 208 | Rituximab | Waldenstrom macroglobulinaemia | O'Connor | National |
| 209 | Sodium Valproate | Schizoaffective disorder | Brunero | NSW |
| 210 | Temazepam | Delirium | Brunero | NSW |
| 211 | Thalidomide | Aphthous stomatitis | Ong | QLD |
| 212 | Thalidomide | Prurigo nodularis | Ong | QLD |
| 213 | Theophylline | Prevention or treatment of apnea of prematurity | O'Donnell | VIC |

NSW: New South Wales; NT: Northern Territory; QLD: Queensland; SA: South Australia; VIC: Victoria; WA: Western Australia
