# Supplementary material for: The extent to which off-patent registered prescription medicines are used for off-label indications in Australia: A scoping review
Source: PLoS One. 2021 Dec 3;16(12):e0261022. doi: 10.1371/journal.pone.0261022 (PMC8641869; doi:10.1371/journal.pone.0261022)
Supplement: S4 Table — (DOCX) [file pone.0261022.s005.docx]

|  | **Off-patent registered prescription medicine** | **Off-label indication reported** | **Extract from QLD LAM showing approved off-label indication (bold text)** | **First author** |
| --- | --- | --- | --- | --- |
| 1 | Clonazepam | Anxiety | Injection 1 mg/mL oral liquid 2.5 mg/mL (0.1 mg/drop. Children too young to swallow tablets; and for a) The prevention of epilepsy; and b) **The treatment of anxiety**, seizures, hiccups and neuropathic pain in patients unable to swallow tablets. | To |
| 2 | Intravenous immunoglobulin | Antibody-mediated rejection | Antithymocyte immunoglobulin rabbit, injection; 25 mg, for use by Transplant Specialists, Oncologists and Haematologists for: a) **Prophylaxis of graft rejection in renal transplantation patients with a history of graft rejection; and b) Treatment of steroid resistant or moderate to severe renal transplant rejection.** | Inglis |
| 3 | Ketamine | Pain | Injection 200 mg/2 mL, (a) For use as a general anaesthetic agent in areas where anaesthesia is delivered; **(b) For use as an analgesic adjuvant under the supervision of an acute pain service, or in emergency departments, where standard analgesics in appropriate doses are insufficient to control pain; (c) For use as an analgesic adjuvant in the treatment of persistent pain under the supervision of specialists in pain medicine and palliative care; (d) For use in the field by practitioners trained to administer ketamine in emergency situations where standard anaesthetic and analgesic agents are contraindicated.** | To |
| 4 | Loratadine | Acute allergic reactions and anaphylaxis | Tablet 10mg, Specialist Staff, Country Medical Superintendents and Endorsed Podiatrists **where other antihistamines are inappropriate.** | Taylor |
| 5 | Lorazepam | Agitation | Tablet 1 mg, (a) use with cancer chemotherapy; (b) use with anaesthetics; (c) use in ICU; (d) **use for acute agitation in mental health settings in line with state-wide guidelines**; (e) Specialist Palliative Care practitioners for sublingual use when swallowing is not possible. and injection 2mg in 1mL with restriction 'For rapid tranquilisation of disturbed behaviour and acute agitation in mental health settings in line with state-wide guidelines. | Brunero |
| 6 | Olanzapine | Delirium/agitation | Orally disintegrating tablet, 5 mg, 10 mg, 20 mg a) For use as per the PBS indications; (b) **For use in acute mental health settings for disturbed behaviour and agitation in line with statewide guidelines**; (c) For use in alcohol and drug withdrawal in line with statewide guidelines. (d) For use by haematologists and oncologists for treatment of breakthrough nausea and vomiting in patients on highly emetogenic chemotherapy, for a maximum of 3 days therapy. | To |
| 7 | Olanzapine | Nausea/vomiting | Orally disintegrating tablet, 5 mg, 10 mg, 20 mg a) For use as per the PBS indications; (b) For use in acute mental health settings for disturbed behaviour and agitation in line with statewide guidelines; (c) For use in alcohol and drug withdrawal in line with statewide guidelines. (d) **For use by haematologists and oncologists for treatment of breakthrough nausea and vomiting in patients on highly emetogenic chemotherapy, for a maximum of 3 days therapy.** | To |
| 8 | Ondansetron | Post-operative nausea and vomiting | Injection 4 mg/2 mL, 8 mg/4 mL General use for TGA approved indications; and for (b) second line therapy in hyperemesis gravidarum. injection 8 mg/4 mL (a) Patients on cytotoxic chemotherapy or radiotherapy as per the PBS indications; (b) **Use for postoperative nausea and vomiting (PONV).** …..Oral liquid 4 mg/5 mL 50mL: For use in children who cannot use any other forms of ondansetron….Orally disintegrating tablet 4 mg, (a) Patients on cytotoxic chemotherapy or radiotherapy as per the PBS indications; (b) **use in postoperative nausea and vomiting (PONV) in (i) adult patients not responsive to alternative oral antiemetic therapy and who cannot use other forms of ondansetron;** and (ii) children; (c) Use within Emergency Departments for acute gastroenteritis in children up to age sixteen years when frequent vomiting is interfering with oral rehydration therapy. It is recommended that the Children's Health Queensland Flowchart - Emergency Management of Children with Acute Gastroenteritis be consulted and followed. ….Tablet 8 mg: For patients on cytotoxic chemotherapy or radiotherapy as per the PBS indications, and tablet 4 mg (a) Patients on cytotoxic chemotherapy or radiotherapy as per the PBS indications; (b) **use in postoperative nausea and vomiting (PONV)** in (i) adult patients not responsive to alternative oral antiemetic therapy; and (ii) children. (c) second line therapy in hyperemesis gravidarum. | Turner |
| 9 | Ondansetron | Gastroenteritis | Injection 4 mg/2 mL, 8 mg/4 mL General use for TGA approved indications; and for (b) second line therapy in hyperemesis gravidarum…..Injection 8 mg/4 mL (a) Patients on cytotoxic chemotherapy or radiotherapy as per the PBS indications; (b) Use for postoperative nausea and vomiting (PONV). and oral liquid 4 mg/5 mL 50mL: For use in children who cannot use any other forms of ondansetron…..Orally disintegrating tablet 4 mg, (a) Patients on cytotoxic chemotherapy or radiotherapy as per the PBS indications; (b) use in postoperative nausea and vomiting (PONV) in (i) adult patients not responsive to alternative oral antiemetic therapy and who cannot use other forms of ondansetron; and (ii) children; **(c) Use within Emergency Departments for acute gastroenteritis in children up to age sixteen years when frequent vomiting is interfering with oral rehydration therapy. It is recommended that the Children's Health Queensland Flowchart - Emergency Management of Children with Acute Gastroenteritis be consulted and followed**. Tablet 8 m: For patients on cytotoxic chemotherapy or radiotherapy as per the PBS indications, and tablet 4 mg (a) Patients on cytotoxic chemotherapy or radiotherapy as per the PBS indications; (b) use in postoperative nausea and vomiting (PONV) in (i) adult patients not responsive to alternative oral antiemetic therapy; and (ii) children. (c) second line therapy in hyperemesis gravidarum. | Taylor |
| 10 | Ondansetron | Nausea and Vomiting of pregnancy | Injection 4 mg/2 mL, 8 mg/4 mL General use for TGA approved indications; and for (b) **second line therapy in hyperemesis gravidarum…**..Injection 8 mg/4 mL (a) Patients on cytotoxic chemotherapy or radiotherapy as per the PBS indications; (b) Use for postoperative nausea and vomiting (PONV). and oral liquid 4 mg/5 mL 50mL: For use in children who cannot use any other forms of ondansetron…..Orally disintegrating tablet 4 mg, (a) Patients on cytotoxic chemotherapy or radiotherapy as per the PBS indications; (b) use in postoperative nausea and vomiting (PONV) in (i) **adult patients not responsive to alternative oral antiemetic therapy and who cannot use other forms of ondansetron**; and (ii) children; (c) Use within Emergency Departments for acute gastroenteritis in children up to age sixteen years when frequent vomiting is interfering with oral rehydration therapy. It is recommended that the Children's Health Queensland Flowchart - Emergency Management of Children with Acute Gastroenteritis be consulted and followed…..Tablet 8 mg For patients on cytotoxic chemotherapy or radiotherapy as per the PBS indications. Tablet 4 mg (a) Patients on cytotoxic chemotherapy or radiotherapy as per the PBS indications; (b) use in postoperative nausea and vomiting (PONV) in (i) adult patients not responsive to alternative oral antiemetic therapy; and (ii) children. (c) **second line therapy in hyperemesis gravidarum**. | Colvin |
| 11 | Posaconazole | Acute myeloid leukaemia | Modified release tablet 100 mg, oral liquid, 40 mg/mL (a) After advice on each individual case by an infectious diseases physician or a clinical microbiologist for treatment of severe invasive fungal infections unable to be treated by standard systemic antifungal therapy; or (b) **As part of an infectious diseases approved protocol for prophylaxis of invasive fungal infections, including both yeasts and moulds, in patients who are at high risk of developing these infections, defined as follows: (i) Neutropenic patients with anticipated neutropenia (an absolute neutrophil count of less than 500 cells per cubic millimetre) for at least 10 days, who are receiving chemotherapy for acute myelogenous leukaemia or myelodysplastic syndrome.** (ii) Graft versus host disease (GVHD) patients with acute GVHD grades II to IV or extensive chronic GVHD, who are receiving intensive immunosuppressive therapy after allogeneic haematopoietic stem cell transplant for up to 6 months. Extension past this time requires individual patient approval.) | Inglis |
| 12 | Posaconazole | Malignant otitis externa | Modified release tablet 100 mg, oral liquid, 40 mg/mL (a) **After advice on each individual case by an infectious diseases physician or a clinical microbiologist for treatment of severe invasive fungal infections unable to be treated by standard systemic antifungal therapy;** or (b) As part of an infectious diseases approved protocol for prophylaxis of invasive fungal infections, including both yeasts and moulds, in patients who are at high risk of developing these infections, defined as follows: (i) Neutropenic patients with anticipated neutropenia (an absolute neutrophil count of less than 500 cells per cubic millimetre) for at least 10 days, who are receiving chemotherapy for acute myelogenous leukaemia or myelodysplastic syndrome. (ii) Graft versus host disease (GVHD) patients with acute GVHD grades II to IV or extensive chronic GVHD, who are receiving intensive immunosuppressive therapy after allogeneic haematopoietic stem cell transplant for up to 6 months. | Inglis |
| 13 | Posaconazole | Myelodysplastic syndrome | Modified release tablet 100 mg, oral liquid, 40 mg/mL (a) After advice on each individual case by an infectious diseases physician or a clinical microbiologist for treatment of severe invasive fungal infections unable to be treated by standard systemic antifungal therapy; or (b) **As part of an infectious diseases approved protocol for prophylaxis of invasive fungal infections, including both yeasts and moulds, in patients who are at high risk of developing these infections, defined as follows: (i) Neutropenic patients with anticipated neutropenia (an absolute neutrophil count of less than 500 cells per cubic millimetre) for at least 10 days, who are receiving chemotherapy for acute myelogenous leukaemia or myelodysplastic syndrome.** (ii) Graft versus host disease (GVHD) patients with acute GVHD grades II to IV or extensive chronic GVHD, who are receiving intensive immunosuppressive therapy after allogeneic haematopoietic stem cell transplant for up to 6 months. Extension past this time requires individual patient approval.) | Inglis |
| 14 | Risperidone | Delirium | Modified release injection 25 mg, 37.5 mg and 50mg restricted for the treatment of a) Schizophrenia for continuation therapy in inpatients, for initiation in inpatients when paliperidone is not clinically suitable, and for outpatient use and b) Maintenance treatment, in combination with lithium or sodium valproate, of treatment refractory bipolar I disorder; and oral liquid 1 mg/mL restricted to 30mL: Specialist psychiatrists and paediatricians for (a) behavioural disturbances; (b) schizophrenia; (c) bipolar disorder; and (d) autism. The PBS indications of risperidone oral solution 1mg per mL, 100mL should be consulted and followed; and tablet 1 mg, 2mg, 3mg, 4mg * For use as per the PBS indications *; and 500 microgram (a) For use as per the PBS indications; (b) **For the symptomatic management of severe behavioural disturbance secondary to delirium when non-pharmacological strategies have been unsuccessful and in consultation with a geriatrician or psychiatrist.** | Brunero |
| 15 | Risperidone | Sundowning/ Delirium | Modified release injection 25 mg, 37.5 mg and 50mg restricted for the treatment of a) Schizophrenia for continuation therapy in inpatients, for initiation in inpatients when paliperidone is not clinically suitable, and for outpatient use and b) Maintenance treatment, in combination with lithium or sodium valproate, of treatment refractory bipolar I disorder; and oral liquid 1 mg/mL restricted to 30mL: Specialist psychiatrists and paediatricians for (a) behavioural disturbances; (b) schizophrenia; (c) bipolar disorder; and (d) autism. The PBS indications of risperidone oral solution 1mg per mL, 100mL should be consulted and followed; and tablet 1 mg, 2mg, 3mg, 4mg * For use as per the PBS indications *; and 500 microgram (a) For use as per the PBS indications; (b) **For the symptomatic management of severe behavioural disturbance secondary to delirium when non-pharmacological strategies have been unsuccessful and in consultation with a geriatrician or psychiatrist.** | Brunero |
| 16 | Rituximab | Membranous glomerulonephritis | Injection 1.4 g/11.7 mL, 100mg/10mL and 500 mg/50 mL For use as per PBS indications for Related Pharmaceutical Benefits used in conjunction with Efficient Funding of Chemotherapy - Section 100 arrangements; (a) * For use in accord with PBS Section 100 indications * OR * For use as per the Efficient Funding of Chemotherapy - Section 100 Arrangements Supplement of the PBS *; (b) On the advice of a haematologist for thrombotic thrombocytopenia purpura (TTP) in patients with suboptimal response to standard treatment (e.g. plasma exchange and high-dose steroids) or with relapsed disease; (c) **Nephrologists for induction of remission, or management of relapse of nephrotic syndrome where the underlying abnormality is confirmed to be membranous nephropathy.** | Inglis |
| 17 | Rituximab | Membranous glomerulonephritis | Injection 1.4 g/11.7 mL, 100mg/10mL and 500 mg/50 mL For use as per PBS indications for Related Pharmaceutical Benefits used in conjunction with Efficient Funding of Chemotherapy - Section 100 arrangements; (a) * For use in accord with PBS Section 100 indications * OR * For use as per the Efficient Funding of Chemotherapy - Section 100 Arrangements Supplement of the PBS *; (b) On the advice of a haematologist for thrombotic thrombocytopenia purpura (TTP) in patients with suboptimal response to standard treatment (e.g. plasma exchange and high-dose steroids) or with relapsed disease; (c) **Nephrologists for induction of remission, or management of relapse of nephrotic syndrome where the underlying abnormality is confirmed to be membranous nephropathy** | O'Connor |
| 18 | Rituximab | Membranous nephritis | Injection 1.4 g/11.7 mL, 100mg/10mL and 500 mg/50 mL For use as per PBS indications for Related Pharmaceutical Benefits used in conjunction with Efficient Funding of Chemotherapy - Section 100 arrangements; (a) * For use in accord with PBS Section 100 indications * OR * For use as per the Efficient Funding of Chemotherapy - Section 100 Arrangements Supplement of the PBS *; (b) On the advice of a haematologist for thrombotic thrombocytopenia purpura (TTP) in patients with suboptimal response to standard treatment (e.g. plasma exchange and high-dose steroids) or with relapsed disease; (c) **Nephrologists for induction of remission, or management of relapse of nephrotic syndrome where the underlying abnormality is confirmed to be membranous nephropathy** | Butterly |
| 19 | Rituximab | Membranous nephropathy | Injection 1.4 g/11.7 mL, 100mg/10mL and 500 mg/50 mL For use as per PBS indications for Related Pharmaceutical Benefits used in conjunction with Efficient Funding of Chemotherapy - Section 100 arrangements; (a) * For use in accord with PBS Section 100 indications * OR * For use as per the Efficient Funding of Chemotherapy - Section 100 Arrangements Supplement of the PBS *; (b) On the advice of a haematologist for thrombotic thrombocytopenia purpura (TTP) in patients with suboptimal response to standard treatment (e.g. plasma exchange and high-dose steroids) or with relapsed disease; (c) **Nephrologists for induction of remission, or management of relapse of nephrotic syndrome where the underlying abnormality is confirmed to be membranous nephropathy** | Wongseelashote |
| 20 | Rituximab | Thrombotic thrombocytopenic purpura | Injection 1.4 g/11.7 mL, 100mg/10mL and 500 mg/50 mL For use as per PBS indications for Related Pharmaceutical Benefits used in conjunction with Efficient Funding of Chemotherapy - Section 100 arrangements; (a) * For use in accord with PBS Section 100 indications * OR * For use as per the Efficient Funding of Chemotherapy - Section 100 Arrangements Supplement of the PBS *; (b) **On the advice of a haematologist for thrombotic thrombocytopenia purpura (TTP) in patients with suboptimal response to standard treatment (e.g. plasma exchange and high-dose steroids) or with relapsed disease;** (c) Nephrologists for induction of remission, or management of relapse of nephrotic syndrome where the underlying abnormality is confirmed to be membranous nephropathy. | Butterly |
| 21 | Rituximab | Thrombotic thrombocytopenic purpura | Injection 1.4 g/11.7 mL, 100mg/10mL and 500 mg/50 mL For use as per PBS indications for Related Pharmaceutical Benefits used in conjunction with Efficient Funding of Chemotherapy - Section 100 arrangements; (a) * For use in accord with PBS Section 100 indications * OR * For use as per the Efficient Funding of Chemotherapy - Section 100 Arrangements Supplement of the PBS *; (b) **On the advice of a haematologist for thrombotic thrombocytopenia purpura (TTP) in patients with suboptimal response to standard treatment (e.g. plasma exchange and high-dose steroids) or with relapsed disease;** (c) Nephrologists for induction of remission, or management of relapse of nephrotic syndrome where the underlying abnormality is confirmed to be membranous nephropathy. | Wongseelshote |
| 22 | Rituximab | Thrombotic thrombocytopenic purpura | Injection 1.4 g/11.7 mL, 100mg/10mL and 500 mg/50 mL For use as per PBS indications for Related Pharmaceutical Benefits used in conjunction with Efficient Funding of Chemotherapy - Section 100 arrangements; (a) * For use in accord with PBS Section 100 indications * OR * For use as per the Efficient Funding of Chemotherapy - Section 100 Arrangements Supplement of the PBS *; (b) **On the advice of a haematologist for thrombotic thrombocytopenia purpura (TTP) in patients with suboptimal response to standard treatment (e.g. plasma exchange and high-dose steroids) or with relapsed disease;** (c) Nephrologists for induction of remission, or management of relapse of nephrotic syndrome where the underlying abnormality is confirmed to be membranous nephropathy. | O'Connor |
| 23 | Rituximab | Thrombotic thrombocytopenic purpure | Injection 1.4 g/11.7 mL, 100mg/10mL and 500 mg/50 mL For use as per PBS indications for Related Pharmaceutical Benefits used in conjunction with Efficient Funding of Chemotherapy - Section 100 arrangements; (a) * For use in accord with PBS Section 100 indications * OR * For use as per the Efficient Funding of Chemotherapy - Section 100 Arrangements Supplement of the PBS *; (b) **On the advice of a haematologist for thrombotic thrombocytopenia purpura (TTP) in patients with suboptimal response to standard treatment (e.g. plasma exchange and high-dose steroids) or with relapsed disease;** (c) Nephrologists for induction of remission, or management of relapse of nephrotic syndrome where the underlying abnormality is confirmed to be membranous nephropathy. | Inglis |

QLD LAM: Queensland Health list of approved medicines
