# Supplementary material for: The extent to which off-patent registered prescription medicines are used for off-label indications in Australia: A scoping review
Source: PLoS One. 2021 Dec 3;16(12):e0261022. doi: 10.1371/journal.pone.0261022 (PMC8641869; doi:10.1371/journal.pone.0261022)
Supplement: S5 Table — (DOCX) [file pone.0261022.s006.docx]

|  | **Off-patent registered prescription medicine** | **Off-label indication reported** | **Extract from SAMF showing approved off-label indication (bold text)** | **First author** |
| --- | --- | --- | --- | --- |
| 1 | Droperidol | Nausea/vomiting | Class: **Other antiemetics; 1mg/1mL Injection** | Brunero |
| 2 | Infliximab | Pyoderma gangrenosum | Class: Other dermatological drugs;100 mg injection; Restrictions on use: **For patients with pyoderma gangrenosum (PG) with moderate to severe disease or mild PG that has failed topical preparation and who are being treated with topical treatments as adjunct to systemic therapy guided by physician judgement meeting the criteria specified in the eligibility form.** | Ong |
| 3 | Infliximab | Pyoderma grangrenosum | Class: Other dermatological drugs;100 mg injection; Restrictions on use: **For patients with pyoderma gangrenosum (PG) with moderate to severe disease or mild PG that has failed topical preparation and who are being treated with topical treatments as adjunct to systemic therapy guided by physician judgement meeting the criteria specified in the eligibility form.** | Inglis |
| 4 | Morphine | Dyspnoea | Class: Opioid analgesics; Morphine Hydrochloride 1mg/1mL Mixture, 5mg/1mL Mixture; Restrictions on use :Severe disabling pain, unresponsive to non-opioid analgesics OR **Palliative patients with dyspnoea** | To |
| 5 | Olanzapine | Delirium/agitation | Class: Antipsychotics (Oral and short acting injections); 2.5mg Tablets, 5mg Orally Disintegrating Tablets (ODT), 7.5mg Tablets, 10mg Orally Disintegrating Tablets (ODT), Restrictions on use: PBS criteria OR Inpatient management of **agitation** according to local protocol ....15mg Orally Disintegrating Tablets (ODT), 20mg Orally Disintegrating Tablets (ODT), Restrictions on use: PBS criteria OR Psychiatry / Psychiatry advice for short term management of **agitation** / behaviour disturbance per protocol, for inpatient use and on discharge for tapering course (usually maximum of one month) (non PBS) OR ....10mg Intramuscular Injection Restrictions on use: Management of **agitation** according to local protocol | To |
| 6 | Olanzapine | Nausea/vomiting | Class: Antipsychotics (Oral and short acting injections); 2.5mg Tablets, 5mg Orally Disintegrating Tablets (ODT), 7.5mg Tablets; Restrictions on use: PBS criteria .....OR **For short term use in palliative care patients with nausea and vomiting refractory to other treatments (non PBS)** Class: Antipsychotics (Oral and short acting injections); 10mg Orally Disintegrating Tablets (ODT); Restrictions on use: PBS criteria .... OR **Prophylaxis or treatment of chemotherapy induced nausea and vomiting (CINV) with moderately/highly emetogenic chemotherapeutic drug regimen where aprepitant is not PBS listed or effective and break-through nausea and vomiting despite optimal primary prevention ...... OR For short term use in palliative care patients with nausea and vomiting refractory to other treatments (non PBS)** | To |
| 7 | Ondansetron | Nausea and Vomiting of pregnancy | Class: 5HT3 Antagonists; 4mg/2mL Injection, 8mg/4mL Injection, 4mg/5mL Syrup, 4mg Orally Disintegrating Tablets (ODT), 8mg Orally Disintegrating Tablets (ODT). Note: **Ondansetron may be considered appropriate as an antiemetic for acute nausea and vomiting** OR Pharmaceutical Benefits Scheme (PBS) criteria. | Colvin |
| 8 | Ondansetron | Post-operative nausea and vomiting | Class: 5HT3 Antagonists; 4mg/2mL Injection, 8mg/4mL Injection, 4mg/5mL Syrup, 4mg Orally Disintegrating Tablets (ODT), 8mg Orally Disintegrating Tablets (ODT). Note: **Ondansetron may be considered appropriate as an antiemetic for acute nausea and vomiting** OR Pharmaceutical Benefits Scheme (PBS) criteria. | Turner |
| 9 | Quetiapine | Aggression | Class: Antipsychotics (Oral and short acting injections); 25mg Tablets, 50mg Slow Release Tablets, 100mg Tablets, 150mg Slow Release Tablets, 200mg Slow Release Tablets, 200mg Tablets, 300mg Slow Release Tablets, 300mg Tablets, 400mg Slow Release Tablets; Restrictions on use: PBS criteria OR Psychiatry / Psychiatry advice for short term management of agitation / **behaviour disturbance** per protocol, for inpatient use and on discharge for tapering course (usually maximum of one month) (non PBS) | Brunero |
| 10 | Quetiapine | Aggression/ agitation | Class: Antipsychotics (Oral and short acting injections) 25mg Tablets, 50mg Slow Release Tablets, 100mg Tablets, 150mg Slow Release Tablets, 200mg Slow Release Tablets, 200mg Tablets, 300mg Slow Release Tablets, 300mg Tablets, 400mg Slow Release Tablets; Restrictions on use: PBS criteria OR Psychiatry / Psychiatry advice for short term management of **agitation / behaviour disturbance** per protocol, for inpatient use and on discharge for tapering course (usually maximum of one month) (non PBS) | Brunero |
| 11 | Quetiapine | Agitation | Class: Antipsychotics (Oral and short acting injections); 25mg Tablets, 50mg Slow Release Tablets, 100mg Tablets, 150mg Slow Release Tablets, 200mg Slow Release Tablets, 200mg Tablets, 300mg Slow Release Tablets, 300mg Tablets, 400mg Slow Release Tablets; Restrictions on use: PBS criteria OR Psychiatry / Psychiatry advice for short term management of **agitation** / behaviour disturbance per protocol, for inpatient use and on discharge for tapering course (usually maximum of one month) (non PBS) | Brunero |
| 12 | Rituximab | Acquired haemophilia | Class: Antineoplastic antibodies; 100mg/10mL injection, 500mg/50mL injection; Restrictions on use: PBS criteria.... **OR Acquired haemophilia A; Note: Acquired haemophilia A - Grandfathered onto High Cost Medicines formulary for this indication (subject to review by SAMEP);** Rituximab has been reviewed by the SA Medicines Evaluation Panel (SAMEP) for the following indications: AAV IMM AIHA ITP Membranous nephropathy EBV reactivation | O'Connor |
| 13 | Rituximab | Acquired haemophilia A | Class: Antineoplastic antibodies; 100mg/10mL injection, 500mg/50mL injection; Restrictions on use: PBS criteria.... **OR Acquired haemophilia A; Note: Acquired haemophilia A - Grandfathered onto High Cost Medicines formulary for this indication (subject to review by SAMEP);** Rituximab has been reviewed by the SA Medicines Evaluation Panel (SAMEP) for the following indications: AAV IMM AIHA ITP Membranous nephropathy EBV reactivation | Wongseelashote |
| 14 | Rituximab | ANCA vasculitis | Class: Antineoplastic antibodies; 100mg/10mL injection, 500mg/50mL injection; Restrictions on use: PBS criteria; ..... **OR ANCA-associated vasculitis (AAV);** Class: Immunosuppressants (rheumatology), 500mg/50mL Injection, 100mg/10mL Injection; Restrictions on use: PBS criteria; ....... **OR ANCA-associated vasculitis (AAV);** | O'Connor |
| 15 | Rituximab | ANCA -associated vasculitis | Class: Antineoplastic antibodies; 100mg/10mL injection, 500mg/50mL injection; Restrictions on use: PBS criteria; ..... **OR ANCA-associated vasculitis (AAV);** Class: Immunosuppressants (rheumatology), 500mg/50mL Injection, 100mg/10mL Injection; Restrictions on use: PBS criteria; ....... **OR ANCA-associated vasculitis (AAV);** | Wongseelashote |
| 16 | Rituximab | Antibody-mediated rejection transplant (lung, cardiac, renal) | Class: Antineoplastic antibodies; 100mg/10mL injection, 500mg/50mL injection; Restrictions on use: PBS criteria; **OR Adult patients for desensitisation prior to ABO and HLA incompatible renal transplant;** Class Immunosuppressants (rheumatology), 500mg/50mL Injection, 100mg/10mL Injection; Restrictions on use: PBS criteria; **OR Adult patients for desensitisation prior to ABO and HLA incompatible renal transplant** | O'Connor |
| 17 | Rituximab | Autoimmune haemolytic anaemia | Rituximab is listed on the Statewide High Cost Medicines Formulary for **autoimmune haemolytic anaemia (AIHA) that is steroid refractory,** | O'Connor |
| 18 | Rituximab | Autoimmune haemolytic anaemia | Rituximab is listed on the Statewide High Cost Medicines Formulary for **autoimmune haemolytic anaemia (AIHA) that is steroid refractory,** | Wongseelashote |
| 19 | Rituximab | Dermatomyositis | Class: Antineoplastic antibodies; 100mg/10mL injection, 500mg/50mL injection; Restrictions on use: PBS criteria ....; **OR Refractory Inflammatory Myositis (IMM) - for initial therapy of myositis which has been refractory to treatment with prednisolone plus at least TWO conventional immunosuppressive medications for a duration of 3 months each;** Class Immunosuppressants (rheumatology), 500mg/50mL Injection, 100mg/10mL Injection; Restrictions on use: PBS criteria; ....**OR Refractory inflammatory myositis (IMM) - for initial therapy of myositis which has been refractory to treatment with prednisolone plus at least TWO conventional immunosuppressive medications for a duration of 3 months each;** | Butterly |
| 20 | Rituximab | Idiopathic thrombocytopaenia purpure | Class: Antineoplastic antibodies; 100mg/10mL injection, 500mg/50mL injection; Restrictions on use:PBS criteria; ....; **OR Immune thrombocytopenia (ITP) refractory to best practice first line therapy, with disease duration < 1 year and not having previously received rituximab for ITP**. An eligibility form must be completed. Class: Immunosuppressants (rheumatology), 500mg/50mL Injection, 100mg/10mL Injection; Restrictions on use: PBS criteria;......**OR Immune thrombocytopenia (ITP) refractory to best practice first line therapy, with disease duration < 1 year and not having previously received rituximab for ITP.** An eligibility form must be completed. | Inglis |
| 21 | Rituximab | Idiopathic thrombocytopenic purpura | Class: Antineoplastic antibodies; 100mg/10mL injection, 500mg/50mL injection; Restrictions on use:PBS criteria; ....; **OR Immune thrombocytopenia (ITP) refractory to best practice first line therapy, with disease duration < 1 year and not having previously received rituximab for ITP**. An eligibility form must be completed. Class: Immunosuppressants (rheumatology), 500mg/50mL Injection, 100mg/10mL Injection; Restrictions on use: PBS criteria;......**OR Immune thrombocytopenia (ITP) refractory to best practice first line therapy, with disease duration < 1 year and not having previously received rituximab for ITP.** An eligibility form must be completed. | Butterly |
| 22 | Rituximab | Immune Thrombocytopenia | Class: Antineoplastic antibodies; 100mg/10mL injection, 500mg/50mL injection; Restrictions on use:PBS criteria; ....; **OR Immune thrombocytopenia (ITP) refractory to best practice first line therapy, with disease duration < 1 year and not having previously received rituximab for ITP**. An eligibility form must be completed. Class: Immunosuppressants (rheumatology), 500mg/50mL Injection, 100mg/10mL Injection; Restrictions on use: PBS criteria;......**OR Immune thrombocytopenia (ITP) refractory to best practice first line therapy, with disease duration < 1 year and not having previously received rituximab for ITP.** An eligibility form must be completed. | Chay |
| 23 | Rituximab | Immune Thrombocytopenia | Class: Antineoplastic antibodies; 100mg/10mL injection, 500mg/50mL injection; Restrictions on use:PBS criteria; ....; **OR Immune thrombocytopenia (ITP) refractory to best practice first line therapy, with disease duration < 1 year and not having previously received rituximab for ITP**. An eligibility form must be completed. Class: Immunosuppressants (rheumatology), 500mg/50mL Injection, 100mg/10mL Injection; Restrictions on use: PBS criteria;......**OR Immune thrombocytopenia (ITP) refractory to best practice first line therapy, with disease duration < 1 year and not having previously received rituximab for ITP.** An eligibility form must be completed. | O'Connor |
| 24 | Rituximab | Immune thrombocytopenic purpura | Class: Antineoplastic antibodies; 100mg/10mL injection, 500mg/50mL injection; Restrictions on use:PBS criteria; ....; **OR Immune thrombocytopenia (ITP) refractory to best practice first line therapy, with disease duration < 1 year and not having previously received rituximab for ITP.** An eligibility form must be completed. Class: Immunosuppressants (rheumatology), 500mg/50mL Injection, 100mg/10mL Injection; Restrictions on use: PBS criteria;......**OR Immune thrombocytopenia (ITP) refractory to best practice first line therapy, with disease duration < 1 year and not having previously received rituximab for ITP.** An eligibility form must be completed. | Wongseelashote |
| 25 | Rituximab | Inflammatory myositis | Class: Antineoplastic antibodies; 100mg/10mL injection, 500mg/50mL injection; Restrictions on use: PBS criteria ....; **OR Refractory Inflammatory Myositis (IMM) - for initial therapy of myositis which has been refractory to treatment with prednisolone plus at least TWO conventional immunosuppressive medications for a duration of 3 months each;** Class Immunosuppressants (rheumatology), 500mg/50mL Injection, 100mg/10mL Injection; Restrictions on use: PBS criteria; ....**OR Refractory inflammatory myositis (IMM) - for initial therapy of myositis which has been refractory to treatment with prednisolone plus at least TWO conventional immunosuppressive medications for a duration of 3 months each;** | Inglis |
| 26 | Rituximab | Membranous glomerulonephritis | Class: Antineoplastic antibodies; 100mg/10mL injection, 500mg/50mL injection; Restrictions on use: PBS criteria; ..... **OR Adults with biopsy proven idiopathic membranous nephropathy that is refractory to standard treatment**. An eligibility form must be completed. Class: Immunosuppressants (rheumatology); 500mg/50mL Injection, 100mg/10mL Injection; Restrictions on use: PBS criteria;..... **OR Adults with biopsy proven idiopathic membranous nephropathy that is refractory to standard treatment.** An eligibility form must be completed. | Inglis |
| 27 | Rituximab | Membranous glomerulonephritis | Class: Antineoplastic antibodies; 100mg/10mL injection, 500mg/50mL injection; Restrictions on use: PBS criteria; ..... **OR Adults with biopsy proven idiopathic membranous nephropathy that is refractory to standard treatment.** An eligibility form must be completed. Class: Immunosuppressants (rheumatology); 500mg/50mL Injection, 100mg/10mL Injection; Restrictions on use: PBS criteria;..... **OR Adults with biopsy proven idiopathic membranous nephropathy that is refractory to standard treatment.** An eligibility form must be completed**.** | O'Connor |
| 28 | Rituximab | Membranous nephritis | Class: Antineoplastic antibodies; 100mg/10mL injection, 500mg/50mL injection; Restrictions on use: PBS criteria; ..... **OR Adults with biopsy proven idiopathic membranous nephropathy that is refractory to standard treatment.** An eligibility form must be completed. Class: Immunosuppressants (rheumatology); 500mg/50mL Injection, 100mg/10mL Injection; Restrictions on use: PBS criteria;..... **OR Adults with biopsy proven idiopathic membranous nephropathy that is refractory to standard treatment. An eligibility form must be completed.** | Butterly |
| 29 | Rituximab | Membranous nephropathy | Class: Antineoplastic antibodies; 100mg/10mL injection, 500mg/50mL injection; Restrictions on use: PBS criteria; ..... **OR Adults with biopsy proven idiopathic membranous nephropathy that is refractory to standard treatment.** An eligibility form must be completed. Class: Immunosuppressants (rheumatology); 500mg/50mL Injection, 100mg/10mL Injection; Restrictions on use: PBS criteria;..... **OR Adults with biopsy proven idiopathic membranous nephropathy that is refractory to standard treatment.** An eligibility form must be completed. | Wongseelashote |
| 30 | Rituximab | Myasthenia gravis | Class: Antineoplastic antibodies; 100mg/10mL injection, 500mg/50mL injection; Restrictions on use: PBS criteria; .......; **OR Adult patients with moderate to severe muscle-specific tyrosine kinase (MuSK) antibody associated myasthenia gravis (MG).** An eligibility form must be completed. Rituximab is not listed on the South Australian Medicines Formulary for non-MuSK antibody associated myasthenia gravis (MG) but is available (via streamlined non-formulary request form) from Neurology for the following: Adult patients with moderate to severe AChR antibody associated MG or seronegative MG who have had an inadequate response to corticosteroids, and who have failed at least two corticosteroid sparing agents. | O'Connor |
| 31 | Rituximab | Myasthenia gravis | Class: Antineoplastic antibodies; 100mg/10mL injection, 500mg/50mL injection; Restrictions on use: PBS criteria; .......; **OR Adult patients with moderate to severe muscle-specific tyrosine kinase (MuSK) antibody associated myasthenia gravis (MG).** An eligibility form must be completed. Rituximab is not listed on the South Australian Medicines Formulary for non-MuSK antibody associated myasthenia gravis (MG) but is available (via streamlined non-formulary request form) from Neurology for the following: Adult patients with moderate to severe AChR antibody associated MG or seronegative MG who have had an inadequate response to corticosteroids, and who have failed at least two corticosteroid sparing agents. | Inglis |
| 32 | Rituximab | Myasthenia gravis | Class: Antineoplastic antibodies; 100mg/10mL injection, 500mg/50mL injection; Restrictions on use: PBS criteria; .......; **OR Adult patients with moderate to severe muscle-specific tyrosine kinase (MuSK) antibody associated myasthenia gravis (MG).** An eligibility form must be completed. Rituximab is not listed on the South Australian Medicines Formulary for non-MuSK antibody associated myasthenia gravis (MG) but is available (via streamlined non-formulary request form) from Neurology for the following: Adult patients with moderate to severe AChR antibody associated MG or seronegative MG who have had an inadequate response to corticosteroids, and who have failed at least two corticosteroid sparing agents. | Butterly |
| 33 | Rituximab | Myasthenia gravis | Class: Antineoplastic antibodies; 100mg/10mL injection, 500mg/50mL injection; Restrictions on use: PBS criteria; .......; **OR Adult patients with moderate to severe muscle-specific tyrosine kinase (MuSK) antibody associated myasthenia gravis (MG).** An eligibility form must be completed. Rituximab is not listed on the South Australian Medicines Formulary for non-MuSK antibody associated myasthenia gravis (MG) but is available (via streamlined non-formulary request form) from Neurology for the following: Adult patients with moderate to severe AChR antibody associated MG or seronegative MG who have had an inadequate response to corticosteroids, and who have failed at least two corticosteroid sparing agents. | Wongseelashote |
| 34 | Rituximab | Myositis (polymyositis/ Inclusion body myositis/ necrotising) | Class: Antineoplastic antibodies; 100mg/10mL injection, 500mg/50mL injection; Restrictions on use: PBS criteria ....; **OR Refractory Inflammatory Myositis (IMM) - for initial therapy of myositis which has been refractory to treatment with prednisolone plus at least TWO conventional immunosuppressive medications for a duration of 3 months each;** Class Immunosuppressants (rheumatology), 500mg/50mL Injection, 100mg/10mL Injection; Restrictions on use: PBS criteria; ....**OR Refractory inflammatory myositis (IMM) - for initial therapy of myositis which has been refractory to treatment with prednisolone plus at least TWO conventional immunosuppressive medications for a duration of 3 months each;** | Chay |
| 35 | Rituximab | Pemphigus | Class: Immunosuppressants (rheumatology); 500mg/50mL Injection, 100mg/10mL Injection; Restrictions on use: PBS criteria;..... **OR Adult patients with pemphigus vulgaris or pemphigus foliaceus who are refractory to conventional immunosuppression or who are steroid-dependent**. An eligibility form must be completed. | O'Connor |
| 36 | Rituximab | Pemphigus vulgaris | Class: Immunosuppressants (rheumatology); 500mg/50mL Injection, 100mg/10mL Injection; Restrictions on use: PBS criteria;..... **OR Adult patients with pemphigus vulgaris or pemphigus foliaceus who are refractory to conventional immunosuppression or who are steroid-dependent.** An eligibility form must be completed. | Inglis |
| 37 | Rituximab | Pemphigus vulgaris | Class: Immunosuppressants (rheumatology); 500mg/50mL Injection, 100mg/10mL Injection; Restrictions on use: PBS criteria;..... **OR Adult patients with pemphigus vulgaris or pemphigus foliaceus who are refractory to conventional immunosuppression or who are steroid-dependent.** An eligibility form must be completed. | Chay |
| 38 | Rituximab | Pemphigus vulgaris | Class: Immunosuppressants (rheumatology); 500mg/50mL Injection, 100mg/10mL Injection; Restrictions on use: PBS criteria;..... **OR Adult patients with pemphigus vulgaris or pemphigus foliaceus who are refractory to conventional immunosuppression or who are steroid-dependent**. An eligibility form must be completed. | Ong |
| 39 | Rituximab | Pemphigus vulgaris | Class: Immunosuppressants (rheumatology); 500mg/50mL Injection, 100mg/10mL Injection; Restrictions on use: PBS criteria;..... **OR Adult patients with pemphigus vulgaris or pemphigus foliaceus who are refractory to conventional immunosuppression or who are steroid-dependent.** An eligibility form must be completed. | Wongseelashote |
| 40 | Rituximab | Polymyositis | Class: Antineoplastic antibodies; 100mg/10mL injection, 500mg/50mL injection; Restrictions on use: PBS criteria ....; **OR Refractory Inflammatory Myositis (IMM) - for initial therapy of myositis which has been refractory to treatment with prednisolone plus at least TWO conventional immunosuppressive medications for a duration of 3 months each;** Class Immunosuppressants (rheumatology), 500mg/50mL Injection, 100mg/10mL Injection; Restrictions on use: PBS criteria; ....**OR Refractory inflammatory myositis (IMM) - for initial therapy of myositis which has been refractory to treatment with prednisolone plus at least TWO conventional immunosuppressive medications for a duration of 3 months each;** | Sharma |
| 41 | Rituximab | Polymyositis/ dermatomyositis/ myositis | Class: Antineoplastic antibodies; 100mg/10mL injection, 500mg/50mL injection; Restrictions on use: PBS criteria ....; **OR Refractory Inflammatory Myositis (IMM) - for initial therapy of myositis which has been refractory to treatment with prednisolone plus at least TWO conventional immunosuppressive medications for a duration of 3 months each;** Class Immunosuppressants (rheumatology), 500mg/50mL Injection, 100mg/10mL Injection; Restrictions on use: PBS criteria; ....**OR Refractory inflammatory myositis (IMM) - for initial therapy of myositis which has been refractory to treatment with prednisolone plus at least TWO conventional immunosuppressive medications for a duration of 3 months each;** | O'Connor |
| 42 | Rituximab | Post-bone marrow transplant epstein-barr virus | Class: Antineoplastic antibodies; 100mg/10mL injection, 500mg/50mL injection; Restrictions on use: PBS criteria....OR **Prevention of post-transplant lymphoproliferative disease (PTLD) in patients with Epstein-Barr Virus (EBV) reactivation or viraemia following allogeneic stem cell transplantation.** An eligibility form must be completed; | O'Connor |
| 43 | Rituximab | Prophylaxis of transplant rejection | Class: Antineoplastic antibodies; 100mg/10mL injection, 500mg/50mL injection; Restrictions on use: PBS criteria; **OR Adult patients for desensitisation prior to ABO and HLA incompatible renal transplant;** Class: Immunosuppressants (rheumatology); 500mg/50mL Injection, 100mg/10mL Injection; Restrictions on use: PBS criteria; **OR Adult patients for desensitisation prior to ABO and HLA incompatible renal transplant** | O'Connor |
| 44 | Rituximab | Renal transplant (B cell-positive cross match) | Class: Antineoplastic antibodies; 100mg/10mL injection, 500mg/50mL injection; Restrictions on use: PBS criteria; **OR Adult patients for desensitisation prior to ABO and HLA incompatible renal transplant;** Class: Immunosuppressants (rheumatology); 500mg/50mL Injection, 100mg/10mL Injection; Restrictions on use: PBS criteria; **OR Adult patients for desensitisation prior to ABO and HLA incompatible renal transplant** | Butterly |

SAMF: South Australian Medicines Formulary
