# Supplementary material for: The extent to which off-patent registered prescription medicines are used for off-label indications in Australia: A scoping review
Source: PLoS One. 2021 Dec 3;16(12):e0261022. doi: 10.1371/journal.pone.0261022 (PMC8641869; doi:10.1371/journal.pone.0261022)
Supplement: S6 Table — (DOCX) [file pone.0261022.s007.docx]

|  | **Off-patent registered prescription medicine** | **Extract from QLD LAM showing approved off-label indication (bold text)** |
| --- | --- | --- |
| 1 | Alteplase | Injection 10 mg, 50 mg ......**c) fibrinolytic management of obstructed IV access**; pre-filled syringe, 1mg in 1mL ....**For prevention of central venous catheter malfunction and central venous catheter associated bacterial infection in children on maintenance haemodialysis who are under the care of Specialist Renal Physicians.** |
| 2 | Azithromycin | Injection 500 mg; On the advice of an infectious diseases physician, clinical microbiologist or in conjunction with an antimicrobial stewardship (AMS) team protocol. Powder for oral liquid (200 mg/5 mL): For use as per the PBS indications for trachoma; and **for the treatment and prophylaxis of laboratory proven or suspected Bordetella pertussis infection in children and infants**. Tablet (500 mg, 600 mg): For treatment of Chlamydia trachomatis where alternative therapy is ineffective or inappropriate; **Cystic fibrosis specialists treating cystic fibrosis patients (six years and older) as prophylaxis of exacerbations of chronic Pseudomonas aeruginosa infection; For the treatment of laboratory proven Bordetella pertussis infection in adults; For the prophylaxis of Bordetella pertussis infection in susceptible contacts of proven cases as advised by an infectious diseases or public health physician; Thoracic physicians for the treatment of Mycobacterium avium complex pulmonary infection in adults.** |
| 3 | Bivalirudin | Injection 250 mg. For patients undergoing non-urgent percutaneous coronary intervention; **On the advice of a haematologist, vascular physician or clinical pharmacologist for use as an alternative anticoagulant therapy for acute management of patients with heparin induced thrombocytopenia (HIT) in patients who have liver impairment.** |
| 4 | Bleomycin | Injection 15 000 international units. **Interventional radiologists and/or surgeons as a first-line sclerotherapy agent for the treatment of microcystic lymphatic malformations and as an alternative sclerotherapy agent for treatment of other subtypes of veno-lymphatic malformations (at the discretion of the treating clinician).** or For use as per the Efficient Funding of Chemotherapy - Section 100 Arrangements Supplement of the PBS or **For treatment of malignant lymphoma or squamous carcinoma unresponsive to any other therapy or, when owing to severe depression of bone marrow, other therapy cannot be used.** |
| 5 | Botulinum toxin type A | Injection, 100 units. a) Specialist Staff for use in accord with PBS Section 100 indications for outpatient or day patient use only **b) Specialist Colorectal Surgeons for the treatment of female patients with chronic anal fissure (where Crohn disease has been excluded) who have failed a two month trial of conservative therapy (including glyceryl trinitrate ointment 0.2%). Maximum of two treatment doses**. **c) Specialist ophthalmologists only for protective ptosis. d) Specialist paediatric surgeons and paediatric gastroenterologists for treatment in children with pain (proctalgia fugax) secondary to anal sphincter spasm or anal fissure refractory to standard treatment with laxatives. e) Gastroenterologists for management of achalasia in patients who are not candidates for surgical myotomy or endoscopic dilatation treatment.** |
| 6 | Cidofovir | Injection 375 mg/5 mL. **Ear, nose and throat surgeons and respiratory physicians for the treatment of recurrent respiratory papillomatosis on the approval of an infectious diseases physician or antimicrobial stewardship team.** |
| 7 | Cyclizine | Injection 50 mg/mL, tablet 50 mg. Paediatric specialist staff for use in children as second line therapy in prevention and treatment of postoperative nausea and vomiting**, and third line therapy in chemotherapy-induced emesis.** |
| 8 | Danaparoid sodium | Injection 750 anti-Xa units/0.6 mL. **On the advice of a haematologist, vascular physician or clinical pharmacologist for use as an alternative anticoagulant therapy for acute management of patients with heparin induced thrombocytopenia (HIT) in patients with a calculated GFR 15 - 30mL/min; or for use in patients with previous HIT who are on haemodialysis. Cross reactivity testing is desirable.** |
| 9 | Enoxaparin sodium | Injection 100 mg/mL, 20 mg/0.2 mL, 40 mg/0.4 mL, 60 mg/0.6 mL, 80 mg/0.8 mL. a) paediatric patients; (b) haemodialysis; **(c) acute coronary syndrome;** (d) treatment of venous thromboembolism (VTE) [Note: Removal of enoxaparin for treatment of VTE remains under active consideration]. |
| 10 | Entecavir | Tablet 1 mg, 500 microgram (a) For use in accord with PBS Section 100 indications; **(b) For use by specialist staff for the prevention of Hepatitis B reactivation in patients with serological evidence of prior infection who are receiving immunosuppressive therapy. Therapy should be in consultation with or managed by a Hepatology or Infectious Diseases Service and follow established protocols for treatment duration, monitoring whilst on therapy and follow up**. |
| 11 | Fentanyl | Injection 100 microgram/2 mL (other forms also available). **For severe disabling pain not responsive to non-narcotic analgesics.** For intranasal use in paediatric patients in the Emergency Department. * CONTROLLED DRUG ** |
| 12 | Fludarabine | Injection, 50 mg, 50 mg/2 mL, tablet 10 mg. For use as per the Efficient Funding of Chemotherapy - Section 100 Arrangements Supplement of the PBS, Intermediate or high risk chronic lymphocytic leukaemia where first line treatment (including either cyclo-phosphamide or chlorambucil) has proven ineffective or is contraindicated OR **For Specialist Haematologists for use in induction and consolidation in relapsed and/or refractory acute myeloid leukaemia, or in patients with contraindication to anthracyclines.** Tablet: General use for TGA approved indications; and **Specialist Haematologists for the treatment of relapsed and/or refractory low grade lymphoma in patients who have received at least 2 alternate prior therapies.** |
| 13 | Fondaparinux | Injection 2.5 mg/0.5 mL (a) Patients with NSTEACS undergoing invasive or medical management (except for those going directly for emergency (<120 min) invasive management [PCI]) ) **(b) On the advice of a haematologist, vascular physician or clinical pharmacologist for use as an alternative anticoagulant therapy for acute management of patients with heparin induced thrombocytopenia (HIT) in patients with a calculated GFR >30mL/min who cannot take oral treatment.** |
| 14 | Ketorolac | Injection 10 mg/mL, 30 mg/mL. For postoperative pain where commonly indicated opioids are best avoided (a) postoperative pain where commonly indicated opioids are best avoided; and **(b) management of migraine or ureteric colic, as a single dose, when oral NSAID therapy is not tolerated or is considered inappropriate.** |
| 15 | Leuprorelin | Modified release injection, 22.5 mg intramuscular depot and subcutaneous depot (other strengths available). For discharge and outpatient use as per the PBS indications; **Paediatric Endocrinologists and Paediatricians under the supervision of a Paediatric Endocrinologist, for pubertal suppression in children and adolescents with persistent gender dysphoria (note that therapy MUST have been initiated by the Statewide CHQ Gender Service); Endocrinologists and Physicians under the supervision of an Endocrinologist for ongoing maintenance of pubertal suppression in patients with persistent gender dysphoria previously initiated by the Statewide CHQ Gender Service.** |
| 16 | Methotrexate | Injection 7.5 mg/0.15 mL, 50 mg/2 mL. For 7.5 mg/0.15 mL: (a) For discharge and outpatient use as per the PBS indications; **(b) For use by, or in consultation with, a paediatric rheumatologist for children with juvenile idiopathic arthritis or non-infectious (inflammatory) uveitis.** For 50 mg/2 mL: * For use as per the Efficient Funding of Chemotherapy - Section 100 Arrangements Supplement of the PBS *or **Specialist Rheumatologists; Specialist Staff for pharmacological management of ectopic pregnancy.** |
| 17 | Misoprostol | Tablet 200 microgram for: (a) reduction in the incidence of gastrointestinal complications in patients who have a history of peptic ulcer disease and in whom NSAID therapy is essential; **(b) second line management of primary post partum haemorrhage;**(c) Specialist Staff for the therapeutic termination of pregnancy and the management of missed abortion. |
| 18 | Moxifloxacin | Injection, 400 mg/250 mL. For use after approval by an Infectious Diseases Physician or a Clinical Microbiologist or in accord with an Infectious Diseases approved protocol: a) For empirical therapy of patients with community acquired pneumonia or **acute bacterial meningitis who have immediate hypersensitivity to penicillin and cannot be given cephalosporin**; OR ......... |
| 19 | Nifedipine | Modified release tablet 20 mg, 30 mg, 60 mg, tablet / capsule 10 mg, 20 mg. **For use in tocolysis (The slowing or halting of labor during the birth process) only.** |
| 20 | Tenofovir with emtricitabine tablet 300mg-200mg (PEP starter pack A) | Tablet Kit containing 3 x tenofovir with emtricitabine tablet 300mg-200mg. **For post exposure prophylaxis in accordance with Queensland Health guidelines on the management of non-occupational exposure to HIV, and occupational exposure to blood and body fluids in conjunction with Post-exposure Prophylaxis for HIV: Australian National Guidelines.** |
| 21 | Rivaroxaban | Tablet 10 mg, 15mg, 20mg. 10mg: For use as per the PBS indications; **or Venous thromboembolism (VTE) prophylaxis for inpatients previously diagnosed with heparin induced thrombocytopenia (HIT)**. 15mg and 20mg: For use as per the PBS indications; or **On the advice of a haematologist, vascular physician or clinical pharmacologist for use as an alternative anticoagulant therapy for acute management of patients with heparin induced thrombocytopenia (HIT) in patients with a calculated GFR >30mL/min**. |
| 22 | Terbutaline | Injection 500 microgram/mL, powder for inhalation 500 microgram/actuation. General use for TGA approved indications; **and for treatment of uterine hyperstimulation / tachysystole.** |
| 23 | Ticagrelor | Dispersible tablet 90mg: a) For inpatient use in combination with aspirin for treatment of STEMI and high risk non-ST elevation acute coronary syndrome; (b) Use by the Queensland Ambulance Service (QAS) in accordance with the QAS Ticagrelor Drug Therapy Protocol; **(c) Interventional radiologists or neurosurgical specialists for inpatient use in proven clopidogrel non-responders identified by the multiplate / VerifyNow P2Y12 assay post neuro-endovascular stent insertion.** Tablet 90: (a) To be used in combination with aspirin for treatment of STEMI and high risk non-ST elevation acute coronary syndrome; **(b) Interventional radiologists or neurosurgical specialists for use in proven clopidogrel non-responders identified by the multiplate / VerifyNow P2Y12 assay post neuro-endovascular stent insertion.** |
| 24 | Tranexamic acid | Injection 1 g/10 mL, 500 mg/5 mL. tablet 500 mg. Specialist Anaesthetists, Specialist Intensivists, Specialist Surgical Staff and Cardiac Perfusionists for: **a) Major haemorrhage with concomitant hyperfibrinolysis,** and b) Prophylaxis of intra- and post-operative bleeding during major surgical procedures where there is a high likelihood of transfusion requirement; or Credentialed Emergency Department Senior Medical Officers **to treat hypotensive trauma patients with uncontrolled haemorrhage, preferably within one hour, but no later than 3 hours after trauma; or For use in severe to life threatening bleeding in line with Qld Department of Health guidelines for managing patients on novel oral anticoagulants; or ENT surgeons, or ED medical officers in consultation with the on-call ENT surgeon, for management of post-tonsillectomy haemorrhage (primary or secondary) in paediatric patients.** |
| 25 | Triamcinolone acetonide | **Intraocular injection 40mg in 1mL. Specialist ophthalmologists for intraocular oedematous and neovascular disease of the eye.** |
| 26 | Ursodeoxycholic acid | Capsule 250 mg, oral liquid 50 mg/mL. capsule: For proven primary biliary cirrhosis; **and for relief of pruritus and improvement of liver function in biochemically proven intrahepatic cholestasis of pregnancy.** |
| 27 | Valaciclovir | Tablet 500 mg.  **a) On the advice of an Infectious Diseases Physician or a Clinical Microbiologist for the treatment of herpes simplex virus in immunocompromised (haematology and oncology) paediatric patients (up to 18 years of age);** OR ........**g) Use in the paediatric population (up to 18 years of age) for: Prophylaxis of viral infection for 12 months following allogeneic bone marrow transplant; Prophylaxis of viral infection for 3 months following autologous bone marrow transplant**. |
| 28 | Zoledronic acid | Injection 4 mg/5 mL, 4 mg/100 mL, 5 mg/100 mL. (a) For use in accord with PBS Section 100 indications; OR **(b) Paediatricians for the treatment of (i) Osteogenesis imperfecta OR (ii) Primary or secondary osteoporosis in paediatrics with evidence of significant skeletal fragility (e.g. vertebral compression fractures or ≥ 2 low trauma long bone fractures).**... |

QLD LAM: Queensland Health list of approved medicines
