# Supplementary material for: The extent to which off-patent registered prescription medicines are used for off-label indications in Australia: A scoping review
Source: PLoS One. 2021 Dec 3;16(12):e0261022. doi: 10.1371/journal.pone.0261022 (PMC8641869; doi:10.1371/journal.pone.0261022)
Supplement: S7 Table — (DOCX) [file pone.0261022.s008.docx]

|  | **Off-patent registered prescription medicine** | **Extract from SAMF showing approved off-label indication (bold text)** |
| --- | --- | --- |
| 1 | Atazanavir | Class: Antiretrovirals - Protease inhibitors; 200mg Capsules, 300mg Capsules; Restrictions on use: ID / Sexual Health Physicians advice as per PBS Section 100 criteria OR I**D / Sexual Health Physicians for HIV PEP in combination with basic two drug regimen (usually tenofovir/emtricitabine) if the source is known to be HIV positive with unknown or detectable viral load (non PBS)** |
| 2 | Clopidogrel | Class: Antiplatelet drugs; 75mg Tablets; Restrictions on use: PBS criteria OR **Vascular Surgeons for one month post: - Peripheral artery stenting- Tibial angioplasty** - PTFE (Synthetic) bypass graft in patients at high risk of graft occlusion (eg. previous native-vein bypass occlusion) OR Paediatric Cardiology- Congenital heart disease with arterial shunts, stents or conduits at high risk of occlusion - Post Kawasaki disease if aspirin is contraindicated OR **Cardiology post TAVI for a duration of up to 6 months OR In combination with aspirin 100mg tablet, Stroke Unit use only for prevention of TIA / Stroke attributable to large artery atherosclerosis in patients at high-risk of recurrence up to 90 days duration only (non PBS)** |
| 3 | Danazol | Class: Drugs for endometriosis; 200mg Capsules; Restrictions on use: Hereditary angio-oedema as per PBS criteria OR **Haematology for myelofibrosis / aplastic anaemia (non PBS)** |
| 4 | Darunavir | Class: Antiretrovirals - Protease inhibitors; 800mg Tablets, 600mg Tablets; Restrictions on use: ID / Sexual Health Physicians advice as per PBS Section 100 criteria OR **ID / Sexual Health Physicians for HIV PEP in combination with basic two drug regimen (usually tenofovir/emtricitabine) if the source is known to be HIV positive with unknown or detectable viral load (non PBS)** |
| 5 | Etanercept | Class: TNF-alpha antagonists (rheumatology); 25mg Injection, 50mg Injection; Restrictions on use: PBS criteria **OR Islet cell transplantation as per approved protocol (non PBS) OR For the management of acute Graft Versus Host Disease, in patients not responding to other immunosuppressant therapy (non PBS)** |
| 6 | Fluvoxamine | Class: Selective serotonin reuptake inhibitors; 50mg Tablets, 100mg Tablets; Restrictions on use: Patients < 18 years for obsessive-compulsive disorder or when fluoxetine not appropriate on advice of paediatrician or child psychiatrist as per PBS criteria OR Adults for obsessive-compulsive disorder as per PBS criteria OR Adults when sertraline not appropriate as per PBS criteria OR **Under psychiatric advice in adults for augmentation of clozapine levels (non PBS)** |
| 7 | Ivabradine | Class: Other antianginal drugs; 5mg Tablets, 7.5mg Tablets; Restrictions on use: Heart failure as per PBS criteria OR **Cardiology for heart rate control prior to CT coronary angiogram, when beta-blockers ineffective or not tolerated (two doses only) (non PBS)** |
| 8 | Lamotrigine | Class: Other antiepileptics; 5mg, 25mg, 50mg, 100mg, 200mg Tablets; Restrictions on use: As per PBS criteria OR **On psychiatry advice for the management of treatment resistant bipolar depression (non PBS)** |
| 9 | Levetiracetam | Class: Other antiepileptics; 100mg/1mL Oral liquid, 250mg, 500mg, 1g (1,000mg) Tablets; Restrictions on use: As per PBS criteria OR **Neurosurgery for post-surgical seizure prophylaxis when other antiepileptic drugs (AED) are not appropriate; for duration of up to 6 weeks post-surgery (non PBS) OR Oncology for seizure prophylaxis where other antiepileptic drugs (AED) are not appropriate due to drug interactions with chemotherapy; for duration of up to 6 weeks post chemotherapy treatment (non PBS).** 500mg/5mL Injection; Restrictions on use: **Management of status epilepticus when failure with other agents OR Neurosurgery OR When oral levetiracetam therapy not appropriate** |
| 10 | Lopinavir + Ritonavir | Class: Antiretrovirals - Protease inhibitors; 200mg-50mg Tablets, 400mg-100mg/5mL Oral Liquid; Restrictions on use: ID / Sexual Health Physicians advice as per PBS Section 100 criteria OR **ID / Sexual Health Physicians for HIV PEP in combination with basic two drug regimen (usually tenofovir/emtricitabine) if the source is known to be HIV positive with unknown or detectable viral load (non PBS)** |
| 11 | Mirtazapine | Class: Other antidepressants; 15mg, 30mg, 45mg Orally Disintegrating Tablets; Restrictions on use: As per PBS Criteria OR **Akathisia in the Forensic setting, on advice or prescribed by a psychiatrist (non PBS)** |
| 12 | Misoprostol | Class: Other drugs for reflux and ulcers; 200microg Tablets Restrictions on use: **Obstetrics and Gynaecology indications (non PBS)** |
| 13 | Ritonavir | Class: Antiretrovirals - Protease inhibitors; 100mg Tablets; Restrictions on use: ID / Sexual Health Physicians advice as per PBS Section 100 criteria OR **ID / Sexual Health Physicians for HIV PEP in combination with a protease inhibitor (usually atazanavir) PLUS a basic two drug regimen (usually tenofovir/emtricitabine) if the source is known to be HIV positive with unknown or detectable viral load (non PBS)** |
| 14 | Zoledronic acid | Class: Bisphosphonates; 5mg/100mL Infusion; Restrictions on use: Outpatients or on discharge: As per PBS criteria OR For treatment of osteoporosis in patients who fulfil PBS criteria for oral bisphosphonates but when oral bisphosphonate therapy not appropriate OR **Endocrinology for management of acute Charcot foot (non PBS) OR Endocrinology or on advice of endocrinology for paediatric patients with osteogenesis imperfecta, other primary or secondary osteoporosis, bone lesions (non PBS)** |

SAMF: South Australian Medicines Formulary
